# Genetic diversity of candidate loci linked to *Mycobacterium tuberculosis* resistance to bedaquiline, delamanid and pretomanid

**DOI:** 10.1038/s41598-021-98862-4

**Published:** 2021-09-30

**Authors:** Paula J. Gómez-González, Joao Perdigao, Pedro Gomes, Zully M. Puyen, David Santos-Lazaro, Gary Napier, Martin L. Hibberd, Miguel Viveiros, Isabel Portugal, Susana Campino, Jody E. Phelan, Taane G. Clark

**Affiliations:** 1 Department of Infection Biology, Faculty of Infectious and Tropical Diseases, London School of Hygiene and Tropical Medicine, Keppel Street, London, UK; 2 Faculdade de Farmácia, Universidade de Lisboa, Lisbon, Portugal; 3 Instituto Nacional de Salud, Lima, Peru; 4 Global Health and Tropical Medicine, GHTM, Instituto de Higiene E Medicina Tropical, IHMT, Universidade Nova de Lisboa, Lisbon, Portugal; 5 Faculty of Epidemiology and Population Health, London School of Hygiene and Tropical Medicine, London, WC1E 7HT UK

**Keywords:** Genetics, Microbial genetics, Bacterial genes

## Abstract

Tuberculosis (TB), caused by *Mycobacterium tuberculosis*, is one of the deadliest infectious diseases worldwide. Multidrug and extensively drug-resistant strains are making disease control difficult, and exhausting treatment options. New anti-TB drugs bedaquiline (BDQ), delamanid (DLM) and pretomanid (PTM) have been approved for the treatment of multi-drug resistant TB, but there is increasing resistance to them. Nine genetic loci strongly linked to resistance have been identified (*mmpR5*, *atpE*, and *pepQ* for BDQ; *ddn*, *fgd1*, *fbiA*, *fbiB*, *fbiC*, and *fbiD* for DLM/PTM). Here we investigated the genetic diversity of these loci across >33,000 *M. tuberculosis* isolates. In addition, epistatic mutations in *mmpL5-mmpS5* as well as variants in *ndh*, implicated for DLM/PTM resistance in *M. smegmatis*, were explored*.* Our analysis revealed 1,227 variants across the nine genes, with the majority (78%) present in isolates collected prior to the roll-out of BDQ and DLM/PTM. We identified phylogenetically-related mutations, which are unlikely to be resistance associated, but also high-impact variants such as frameshifts (e.g. in *mmpR5*, *ddn*) with likely functional effects, as well as non-synonymous mutations predominantly in MDR-/XDR-TB strains with predicted protein destabilising effects. Overall, our work provides a comprehensive mutational catalogue for BDQ and DLM/PTM associated genes, which will assist with establishing associations with phenotypic resistance; thereby, improving the understanding of the causative mechanisms of resistance for these drugs, leading to better treatment outcomes.

## Introduction

*Mycobacterium tuberculosis* (*Mtb*) remains one of the deadliest single infectious agent, leading to 10 million human tuberculosis (TB) cases and 1.4 million associated deaths in 2019^1^. Most TB cases are found in Asia, Africa, and Western Pacific regions. Drug resistance is one of the major threats to control the disease, especially *Mtb* resistant to rifampicin (RR-TB), and multi-drug resistant (MDR-TB; isoniazid and rifampicin). MDR-TB with further resistance to at least one fluoroquinolone and second-line injectable drug has been defined as extensively drug resistant *Mtb* (XDR-TB), but the definition has recently changed, in part due to a need to include bedaquiline (BDQ) and linezolid (LNZ)^2^. More than 3% of new TB cases are RR- or MDR-TB, and among MDR-TB, more than 6% are XDR-TB. In 2019, approximately half a million people developed MDR-TB, and ~ 12,000 patients had XDR-TB^1^.

BDQ, delamanid (DLM) and pretomanid (PTM) comprise the most recent additions to the anti-TB drug armamentarium and therefore constitute alternative effective drugs for resistant cases^3^. BDQ has been in use since 2013^1^, and is a diarylquinoline that inhibits the proton pump ATP synthase, more specifically, the subunit c encoded by the *atpE* gene (*Rv1305*)^4^. DLM is a nitro-dihydro-imidazooxazole derivative that targets the synthesis of the cell wall mycolic acids. It is a pro-drug that is activated by the enzyme deazaflavin dependent nitroreductase encoded by the *ddn* gene (*Rv3547*)^5^, which requires the F_420_ coenzyme system for its activity. DLM started to be used to treat MDR-TB patients in 2014^6^. By the end of 2018, more than fifty countries were using BDQ and DLM. However, resistance to BDQ and DLM emerged quickly, with reports of resistance in vitro^7,8^ and then clinically^9,10^, as well as reported cross-resistance between BDQ and the repurposed antimycobacterial drug clofazimine (CFZ)^11^. There are fears for wider emergence and spread of drug-resistant *Mtb* to these new drugs, particularly among MDR-/XDR-TB strains, which will impose new obstacles that threaten global TB control. PTM was introduced in 2019 in a joint regimen with BDQ and LNZ^1^.

Acquired drug resistance in *Mtb* is almost exclusively due to spontaneous mutations, including single nucleotide polymorphisms (SNPs) and insertions and deletions (indels), in genes coding for drug-targets or drug-converting enzymes^12^. Acquisition and accumulation of resistance conferring mutations sometimes entails fitness loss, which triggers putative compensatory mechanisms^13,14^. Drug resistance can be determined by phenotypic or genotypic methods, and new mutations are being found using genome-wide association and convergent evolution studies^13^. Putative molecular markers of resistance to BDQ include mutations in the drug target *atpE*, and off-target mutations in *mmpR5* (*Rv0678*) and *pepQ* (*Rv2535c*). The *mmpR5* gene encodes for a transcriptional repressor of the MmpS5-MmpL5 efflux pump, whose upregulation has been associated with BDQ resistance^8^. Loss of function of MmpR5 leads to the de-repression of this efflux pump, thereby mediating increased values of minimum inhibitory concentrations (MICs) for BDQ. Some mutations in *mmpR5* have been observed in isolates that pre-date the introduction of BDQ, and may be linked to the use of CFZ or other azoles for fungal infections^15,16^. Epistatic interactions through loss of function mutations in *mmpL5* that counteract the effect of *mmpR5* mutations have been suggested^17,18^. Resistance caused by mutations in the peptidase encoded by *pepQ* has also been reported with increased BDQ MIC values^19^; but the exact mechanism is unclear. Other off-target genes investigated for BDQ resistance include *Rv1979c*, *atpB* and *ppsC*, but only *mmpR5* and *pepQ* have strong experimental evidence of developing mutations under drug exposure in vitro or in vivo^19,20^.

As pro-drugs, the nitroimidazoles DLM and PTM require activation by the deazaflavin (F_420_)-dependent nitroreductase Ddn. Mutations in the essential genes required for the F_420_ cofactor biosynthesis and recycling, including *ddn*, *fgd1*, *fbiA*, *fbiB*, *fbiC*, and *fbiD*, are putative resistance markers that directly hamper DLM/PTM activation or, work indirectly through F_420_ depletion^5,21–23^. Important residues for the interaction of Ddn-PTM are known, which may differ from those involved in Ddn-DLM activation^24^. The role of Fgd1 as a F_420_-dependent glucose-6-phosphate dehydrogenase is to reduce F_420_, which is essential for the correct performance of Ddn. FbiA, FbiB and FbiC are also proteins involved in the activation of DLM and PTM through their role in the synthesis of F_420_ cofactor. Mutations in these 3 genes have been shown to alter the production of F_420_^22^. Similarly, it has been recently demonstrated the essential role of FbiD for the biosynthesis of F_420_ and thereby its participation in DLM and PTM resistance^23^. The contribution of *ndh*, a NADH dehydrogenase, in isoniazid and ethionamide resistance involves retaining an appropriate NADH/NAD^+^ ratio that enables the formation of adducts with NAD^+^, necessary for their activity^25^. The same mechanism of adduct formation has been recently suggested for DLM, with evidence of increased MIC values in *ndh* mutants in a *M. smegmatis* model^26^.

For phenotypic derived resistance, BDQ and DLM drug susceptibility testing use provisional critical concentration values defined by the WHO or the European Committee on Antimicrobial Susceptibility Testing (EUCAST), where the thresholds are highly variable and/or limited^27^. There is currently no established MIC cut-offs for PTM and BDQ by the EUCAST reference method, but ongoing work is attempting to establish these^28,29^. Studies involving genetic-phenotypic functional analysis for resistance have been of limited sample size, and those looking at candidate region genomic variation have considered small numbers of populations. To provide a global view, we perform an analysis of nine candidate genes and their mutations associated with BDQ (*atpE*, *mmpR5* and *pepQ*) and DLM/PTM (*ddn*, *fgd1*, *fbiA*, *fbiB*, *fbiC* and *fbiD*) resistance in > 33,000 clinical *Mtb* isolates, sourced from all WHO regions, and with whole genome sequencing data. In addition, we investigated potential epistatic mutations in *mmpL5* and *mmpS5*, as well as variants in *ndh*. Our goal was to establish the frequency of putative resistance markers across geographical regions and, where possible, rule in or out putative mutations based on source population and date of DLM and BDQ roll-out, individual drug-resistance profiles and phenotypic data, and application of phylogenetic methods and protein structural modelling. *In lieu* of large-scale studies with phenotypic susceptibility testing, we present evidence for mutations involved in BDQ and DLM/PTM putative genotypic resistance, where possible validated by quantitative data on resistance levels. Ultimately, we aim to present a variant catalogue with important mutations that could potentially reduce BDQ, DLM and PTM drug effectiveness globally.

## Results

### The samples

Our study consists of 33,675 publicly available *Mtb* isolates with complete whole-genome sequencing data, collected between 1991 and 2018 across 114 countries^30^. These strains represent the main *Mtb* complex lineages, with the majority in lineage 4 (52%), followed by lineages 2 (25%), 3 (11%) and 1 (10%). Using genotypic resistance prediction^31^, the majority of strains (65%) were pan-susceptible, while 22% were at least MDR-TB , with the remainder being non-MDR but resistant to at least one drug (termed “other drug resistance”) (S1 Table). The vast majority (91%) of isolates were collected before the roll out of BDQ and DLM, and we have used the definition of XDR-TB before the recent WHO update. The most represented geographical areas were Europe and Central Asia, followed by Sub-Saharan Africa, East Asia, and Pacific regions. The highest proportion of MDR-TB strains were from the Latin American and Caribbean region (63%) (S1 Table).

### Mutational diversity and prevalence across resistance associated genes

**Figure 1:**
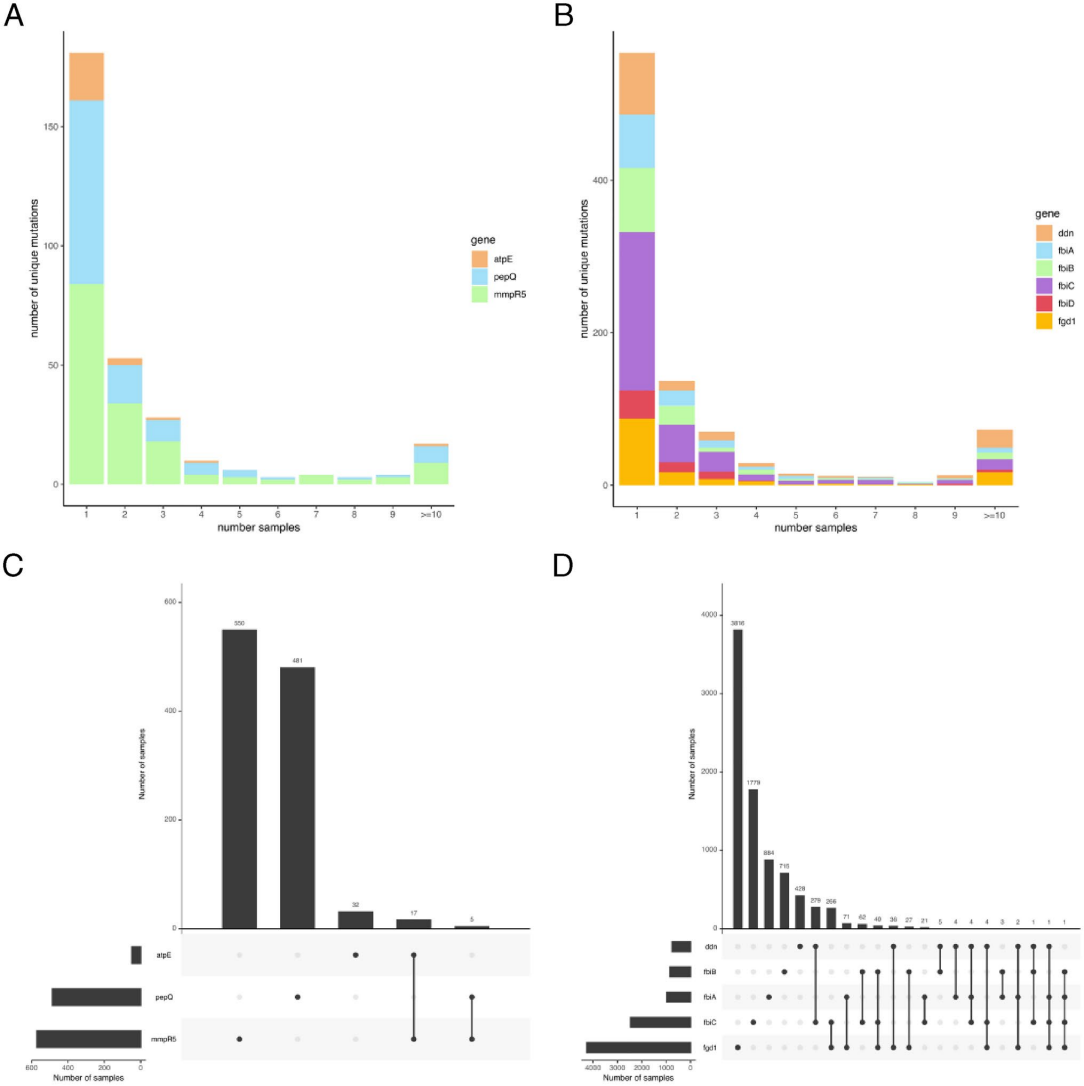
(**A**), (**B**) Frequency of mutations identified across data set. The vertical axis is the number of mutations that are found in 1 to 10 or more isolates (horizontal axis). Colours represent the different genes, each bar showing the distribution of those mutations in the candidate genes for each drug (A = Bedaquiline (BDQ), B = Delamanid (DLM)/Pretomanid (PTM)). (**C**), (**D**) Intersection of mutations in the different genes by sample. Bars represent the number of samples that hold mutations in each gene, or combination of them (horizontal bars show total samples with mutations in each gene); C = BDQ, D = DLM/PTM.

Across the three BDQ resistance candidate genes (*atpE*, *mmpR5* and *pepQ*), we observed 467 unique variants, and focused the analysis on the 309 non-synonymous or indel mutations, distributed across 1,085 (3%) isolates representing all geographical regions and lineages (except lineage 7) (Table 1, S1 Table). Synonymous mutations changing the start codon of *mmpR5* or *pepQ* were not identified. Co-occurrence of multiple mutations in the same candidate gene in an isolate was rare (2% of isolates (n = 22) with > 1 mutation; maximum of 3). Similarly, only 2% (n = 22) of isolates had a mutation in 2 of the 3 BDQ candidate genes (Fig. 1). Most mutations were found in *mmpR5* (n = 163, 53%) and *pepQ* (n = 120, 39%) loci, and the majority of indels (29/33) were present in the former and lead to a high proportion of frameshifts (25/29) (Table 1). Nucleotide diversity in the coding regions of *atpE* was slightly lower than in *mmpR5* and *pepQ* (S2 Figure). The distribution of variants along the *mmpR5* and *pepQ* genes was broadly uniform, but the *atpE* promoter region has a high density of mutations (n = 10, 39% of total mutations in *atpE*), especially between 28 and 41 bp upstream (n = 8, 80% of promoter mutations in *atpE*) (Table 1; S2 Table). In the case of *mmpR5*, there was a greater risk of mutations in MDR-/XDR-TB isolates (adjusted odds ratio > 3.7; *P* < 0.0001), as well as those sourced after year 2014 (adjusted odds ratio 2.574, *P* = 0.002) (Table 1, S2 Table). Most of the BDQ candidate variants (n = 180, 58%) were unique mutations, present in single isolates across the whole data set. Only 17 (6%) of the mutations found in BDQ candidate genes occurred in 10 or more samples (Fig. 1). Of 144 mutations identified previously as associated with increments in MICs (S3 Table), 33 (23%) were identified in our ~33,000 *Mtb *dataset.

**Table 1:** Number of variants per analysed gene across the 33,675 isolates, with the average number of mutations per sample and by resistance profile.

Gene	Drug	Gene SNPs [Indels,fs*]	Prom. SNPs [Indels]	Total analysed [# known**]	# samples with 1 [> 1] mutations	Lineages	Ave. mut. Susc. samples	Ave. mut. MDR samples	Ave. mut. XDR samples	Ave. mut. DR samples	Diversity × 10^−5^***
*atpE*	BDQ	15 [1,0]	5 [5]	26 [1]	48 [1]	1–4, *bov*	0.002	0	0	0.002	0.87
*mmpR5*	BDQ	116 [25, 29]	14 [4]	163 [38]	555 [17]	1–6, *bov*	0.008	0.040	0.079	0.017	3.2
*pepQ*	BDQ	117 [2, 3]	0 [0]	120 [0]	482 [4]	1–6	0.018	0.010	0.004	0.009	2.4
*fgd1*	DLM/PTM	118 [4, 9]	11 [1]	139 [4]	4229 [35]	1–7, *bov*	0.141	0.095	0.075	0.124	23
*ddn*	DLM/PTM	86 [16, 27]	18 [2]	132 [31]	743 [21]	1–5	0.025	0.019	0.015	0.023	7.6
*fbiA*	DLM/PTM	113 [2, 3]	3 [0]	119 [4]	991 [0]	1–5, *bov*	0.037	0.016	0.007	0.019	5.5
*fbiB*	DLM/PTM	135 [1]	0 [0]	136 [3]	851 [3]	1–6, *bov*	0.025	0.022	0.012	0.033	3.6
*fbiC*	DLM/PTM	280 [9, 17]	26 [4]	326 [4]	2413 [45]	1–6, *bov*	0.079	0.052	0.058	0.091	3.8
*fbiD*	DLM/PTM	57 [0,0]	9 [0]	66 [0]	223 [2]	1–4, *bov*	0.008	0.004	0.004	0.007	1.8

Indels = insertions and deletions; DLM = Delamanid; PTM = Pretomanid; BDQ = Bedaquiline; Prom. = promoter; Susc. = Susceptible; DR = Other drug resistance; fs = frame shifts; bov = *M. bovis*; * number of indels that lead to frameshifts; ** see S3 Table; *** Nei’s Pi nucleotide diversity per site (only non-synonymous SNPs considered).

Across the six DLM/PTM candidate genes (*ddn*, *fgd1*, *fbiA*, *fbiB*, *fbiC* and *fbiD*), we observed 1,595 unique mutations, and focused the analysis on 918 (58%) non-synonymous or indel variants found within 8,622 isolates (26% of the samples, all lineages present) (Table 1). Synonymous mutations changing the start codon of the genes starting with amino acids V or L were not identified among our isolates. The *fbiC* gene, which is the largest of the loci considered, accounted for the highest number of different mutations (n = 326, 36% of the total variants identified), with a high density of variants in the promoter region compared to the rest of the coding area (S3 Figure). However, *fgd1* was the most polymorphic gene per isolate, accounting for the higher nucleotide diversity when compared to the other genes (Table 1). The *ddn*, *fgd1* and *fbiD* genes also harboured more than 8% of their variants in the intergenic promoter region. Both *ddn and fbiC* harboured a higher number of indels (44/57) along the whole coding region, compared to the other genes (13/57), where more than half (56%) led to a frameshift. For the six genes, the average number of mutations per sample among susceptible isolates was higher than in MDR- or XDR-TB, which could be due to a higher representation of the different sub-lineages among susceptible samples, or the effects of clonality. For the *ddn* gene, there was a marginally greater risk of mutations in MDR-/XDR-TB isolates (adjusted odds ratio > 1.5; *P* < 0.02; S2 Table). Co-occurrence of variants in genes in the same sample was rare (83 (1%) samples with > 1 mutation; maximum of 3 mutations) (Fig. 1). Likewise, only 828 (10%) isolates with mutations in DLM/PTM candidate genes had at least one mutation in two or more of the genes considered (Fig. 1), where the most prevalent combination of mutations involved *fbiC* with either *ddn* or *fgd1*. A total of 117 (13%) mutations were present at higher frequencies (> 5 samples; note, 62 (7%) mutations with > 10 samples). Of 198 mutations reported previously as associated with some degree of resistance (S3 Table), only 26 associated with DLM or PTM were in our dataset. Co-occurrence of mutations in at least one BDQ and one DLM/PTM candidate gene was also rare, with only less than 2% (n = 153/9,538) of samples harbouring these variants.

Eight mutations in candidate genes (7 DLM/PTM, 1 BDQ) were considered as phylogenetic deep branching variants at high frequency within single sub-lineages (> 50% allele frequency) (S4 Table). Isolates harbouring each of these mutations were collected from > 10 different countries and had a high pairwise SNP distance (> 200). All eight mutations were mostly found in susceptible samples and, where available, date of collection pre-dated the introduction of BDQ and DLM. Seven of these mutations have been previously reported as phylogenetically-related or -informative^17^. These strain specific mutations have been incorporated within the TB-Profiler tool^31^. Nonetheless, the 326 (27%) mutations detected in > 1 isolates and a single homoplastic distribution may denote potentially advantageous polymorphisms with impact at the phenotypic level.

### Diversity and phylogenetic distribution of BDQ-associated variants

**Figure 2:**
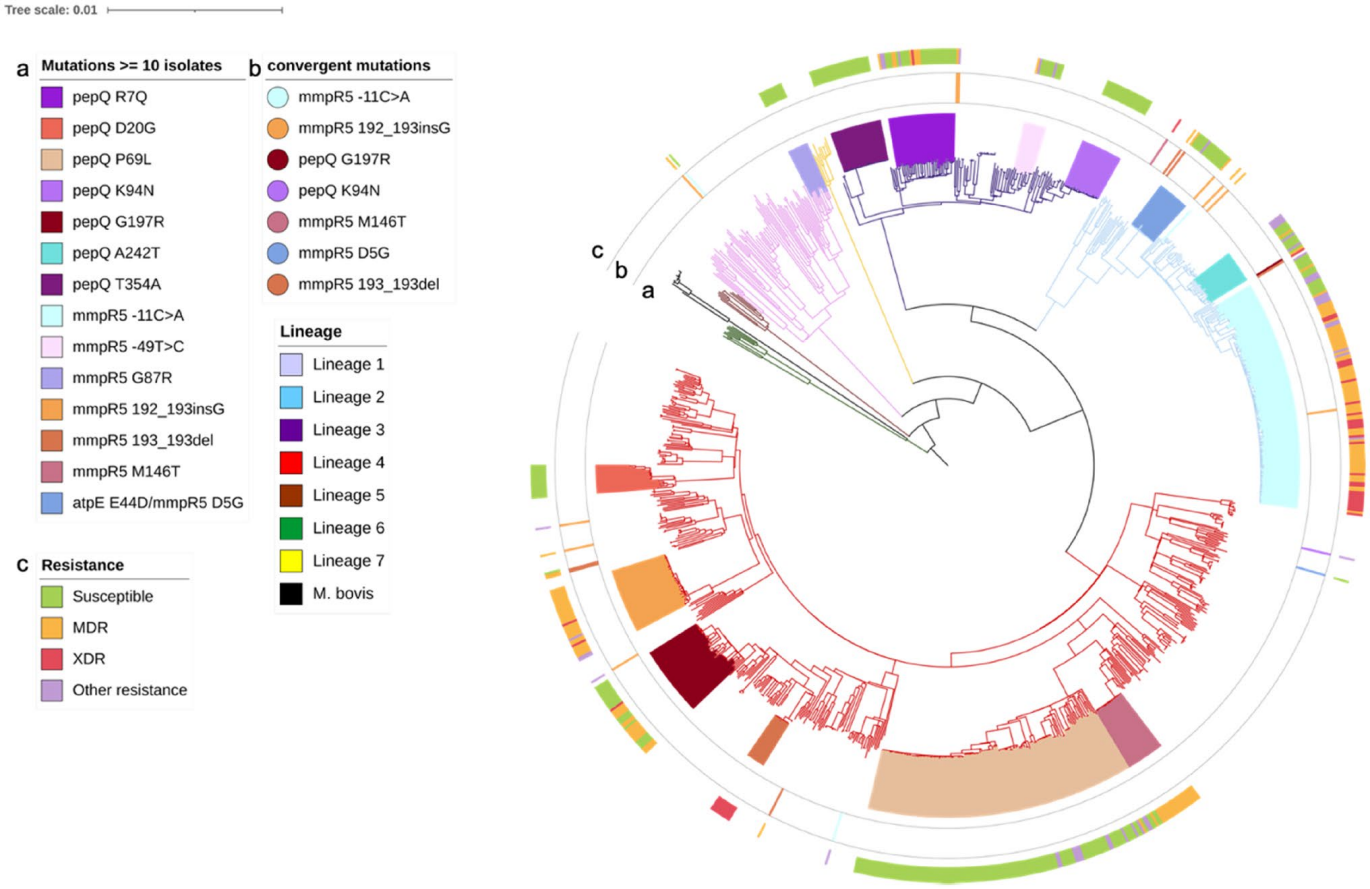
Phylogenetic tree of high frequency (≥ 10 isolates) mutations in bedaquiline candidate genes. The outer track (**c**) shows the resistance phenotype; the second track (**b**) shows the convergent mutations that have arisen in more than one clade; the third track (**a**) shows the clades formed by isolates harbouring the same phylogenetic-related mutations. Branches are coloured by lineage as per legend.

Twenty-two of the 37 most frequent mutations (> 5 isolates, S5 Table) were present in isolates in a single monophyletic cluster. Two mutations (*pepQ* T354A, *mmpR5* M146T) were present in isolates within potential transmission chains (maximum of 11 SNPs difference) (Fig. 2). The majority (13/15) of mutations that showed evidence of convergent evolution were observed in *mmpR5*, of which 8 have been previously associated with increased MICs (Table 2, S3 Table), including 6 variants in high frequency (> 80%) in MDR-/XDR-TB clinical isolates. Two mutations in *mmpR5* (−11C > A, D5G) not linked to in vitro resistance (S3 Table) were also found in high frequency among our isolates, where intergenic −11C > A was prevalent in MDR-/XDR-TB isolates. The −11C > A mutation has been reported in hyper-susceptible strains^15^. The two other high frequency mutations (2/15) in multiple lineages were found to occur in the *pepQ* (G197R, K94N) gene, and predominantly in susceptible strains with one (G197R) predicted to have functional effects by Provean and SNAP2 scores.

**Table 2:** Mutations in bedaquiline candidate genes occurring in at least 5 samples and more than one independent clade.

Mutation	Gene	Freq	Sub-lineage (# isolates)	# sub-lineages	Max SNP dist.*	# Independent Occurrences	Susc. %	MDR or XDR %	Pre-2014%**	Functional support***
**-11C > A**	*mmpR5*	124	2.2.1(122); 4.3.2.1(1); 1.1.1(1)	3	207	3	12.1	76.6	93.1	-
192_193insG (I67fs)	*mmpR5*	44	4(34); 2.2.1(4); 3(2); 4.9(1); 4.8(1); 4.5(1); 1.1.1(1)	7	60	10	0	86.4	100	-
**G197R**	*pepQ*	38	4.3.4.1(37); 2.2.1(1)	2	168	2	52.6	47.4	72.2	S,P
**K94N**	*pepQ*	23	3.1.1(22); 4.1.2(1)	2	24	2	95.7	0	100	-
M146T	*mmpR5*	21	4.4.1.1(20); 2.2.2(1)	2	11	2	0	100	-	S,M
**D5G**	*mmpR5*	18	2.2.1(17); 4.1.2.1(1)	2	33	2	94.4	0	75.0	-
193_193del (I67fs)	*mmpR5*	16	4.3.4.2(10); 2.2.1(3); 4.7(2); 4.3.3.1(1)	4	17	5	0	100	83.3	-
141_142insC	*mmpR5*	15	2.2^+^(8); 4.1.2^+^(2); 4.3^+^(2); 4.4.1.1(1); 3(2)	8	-	11	6.7	86.7	85.7	-
**V20A**	*mmpR5*	10	4.1.2.1(8); 4.3.2.1(1); 2.2.1(1)	3	23	3	90	10	83.3	M
L117R	*mmpR5*	9	3(5); 4.3.4.2(2); 4.2.2(1); 4.1(1)	4	98	5	44.4	44.4	100	S
**L32S**	*mmpR5*	8	2.2.1(8)	1	21	3	0	87.5	50	S,M
G121R	*mmpR5*	7	2.2.2(5); 3(1); 4.4.1.1(1)	3	4	3	0	100	100	S,P
**D141H**	*mmpR5*	7	2.2.1(6); 1.1.3(1)	2	130	2	14.3	57.1	100	S,P
R90C	*mmpR5*	7	2.2.1(6); 4.1.1.3(1)	2	24	4	85.7	0	50	-
N98D	*mmpR5*	5	4.1.2.1(2); 4.4.1.1(2); 2.2.1(1)	3	5	3	0	80	100	-

Sub-lineages: ^+^ = more than 1 sub-lineage; # = number; * Maximum SNP distance calculated in clades of ≥ 5 isolates; Drug resistance (%): Susc. = Susceptible; ** % of number of samples pre-2014/total number of samples with available collection date; *** Functional support: S = snap2 score ≥ 50; P = Provean Score ≤ −4; M = mCSM predicted stability change (ΔΔG) below − 2. Mutations associated with increased MIC for BDQ in previous studies in **bold**; mutations associated with susceptibility to BDQ underlined (see S3 Table).

### atpE and pepQ

Most mutations in *atpE* (20/26; 77%) were found in single isolates (S7 Table), and those with higher frequencies did not show evidence of convergent evolution, being part of single clades (S5 Table, Fig. 2). Of twenty-five novel mutations found in *atpE*, 15 were non-synonymous SNPs, of which 9 were predicted to confer resistance using SUSPECT-BDQ software^32^ (S5 Table, S7 Table). Only the I66V mutation is present in residues involved in BDQ-*atpE* interactions (S4 Figure). The E44D mutation, predicted as conferring resistance, was present in 17 mostly pan-susceptible Beijing (lineage 2.2.1) isolates.

The 120 novel mutations identified in *pepQ* included 117 non-synonymous SNPs (S5 Table) and 3 indels, 2 of them leading to frameshifts found in single isolates (S7 Table, S5 Figure). These frameshifts are likely to be involved in the functional loss of *pepQ*, consistent with others that have been found (see S3 Table). In the absence of a crystal structure of PepQ, SNAP2 and Provean scores revealed 9 mutations with a potential functional effect (S5 Table), and 3 were present in MDR-/XDR-TB isolates.

### mmpR5 mutations

Of the 163 mutations (116 non-synonymous SNPs, 29 indels and 18 promoter variants) found in *mmpR5*, 32 and 14 have been previously associated with MIC incrementation or no change, respectively (S3 Table). A high density of variants (n = 64) in the DNA binding domain was observed, including 14 frameshifts (S6 Figure). In addition, 3 SNPs were translated into stop codons (E13*, W42* and R156*; S5 Table; S7 Table), which are likely to alter the protein function. Three frameshifts (192_193insG, 193_193del, 141_142insC) have a high number of independent occurrences (range: 5–11) in a phylogenetic tree (Table 2, Fig. 2), all previously associated with higher MICs in vitro to BDQ^33^. The 192_193 indel (sometimes denoted as I67fs), involving a premature stop codon, appears in 44 isolates through 10 independent acquisitions. The largest subclade (34 isolates) consists of resistant lineage 4 strains, with all except one sourced from Peru and collected between years 2009 and 2012, prior to the introduction of BDQ in that country (Table 2; S7 Figure). A potential epistatic effect involving the 605_605 deletion in *mmpL5* was found in 33 of these isolates, confirming recent work^17,18^. In addition, two isolates from Malawi belonging to lineage 4.3.4.2.1 with a pan-susceptible profile had the beginning and most of *mmpR5* deleted (778866_779429del), which could have similar epistatic effects.

The *mmpR5* 193_193 deletion (I67fs) was present in XDR-TB isolates from Portugal (lineage 4.3.4.2; n = 10) and present in the phylogenetic tree an additional 4 times independently in modern strains within different sub-lineages (Table 2, Fig. 2). To investigate the contribution of this mutation to BDQ resistance levels, we screened for it in a recently published dataset focused on the evolutionary history of MDR-TB in Portugal^34^. One clinical isolate (MTB1) was available with a BDQ MIC value of 0.25 mg/L, which is at least 6- to 8-fold higher in comparison to wild-type strains, including one isolate from the same phylogenetic clade and *M. tuberculosis* H37Rv (ATCC 27,294) (S9 Table). CFZ MICs determined in parallel showed a 4- to 6-fold increase for the *mmpR5* mutant strain, which corroborates the high impact of this variant on MmpR5 function, and is consistent with previous findings in South Africa^16^. Further, our analysis confirmed the presence of the *mmpR*5 M146T mutation within a transmission cluster in Eswatini^35^, as well as in an independent XDR-TB (lineage 2.2.2) strain (Table 2, Fig. 2). Twenty-one of the remaining SNPs in *mmpR5*, including high frequency D5G, V20A, L117R, L32S, G121R, D141H, R90C and N98D, were in the same residue where mutations associated with increments in MIC have been observed; however, mutations V20A and D141H have associated MIC values within a susceptibility range^36^.

### Mutational diversity in Delamanid and Pretomanid associated genes

**Figure 3:**
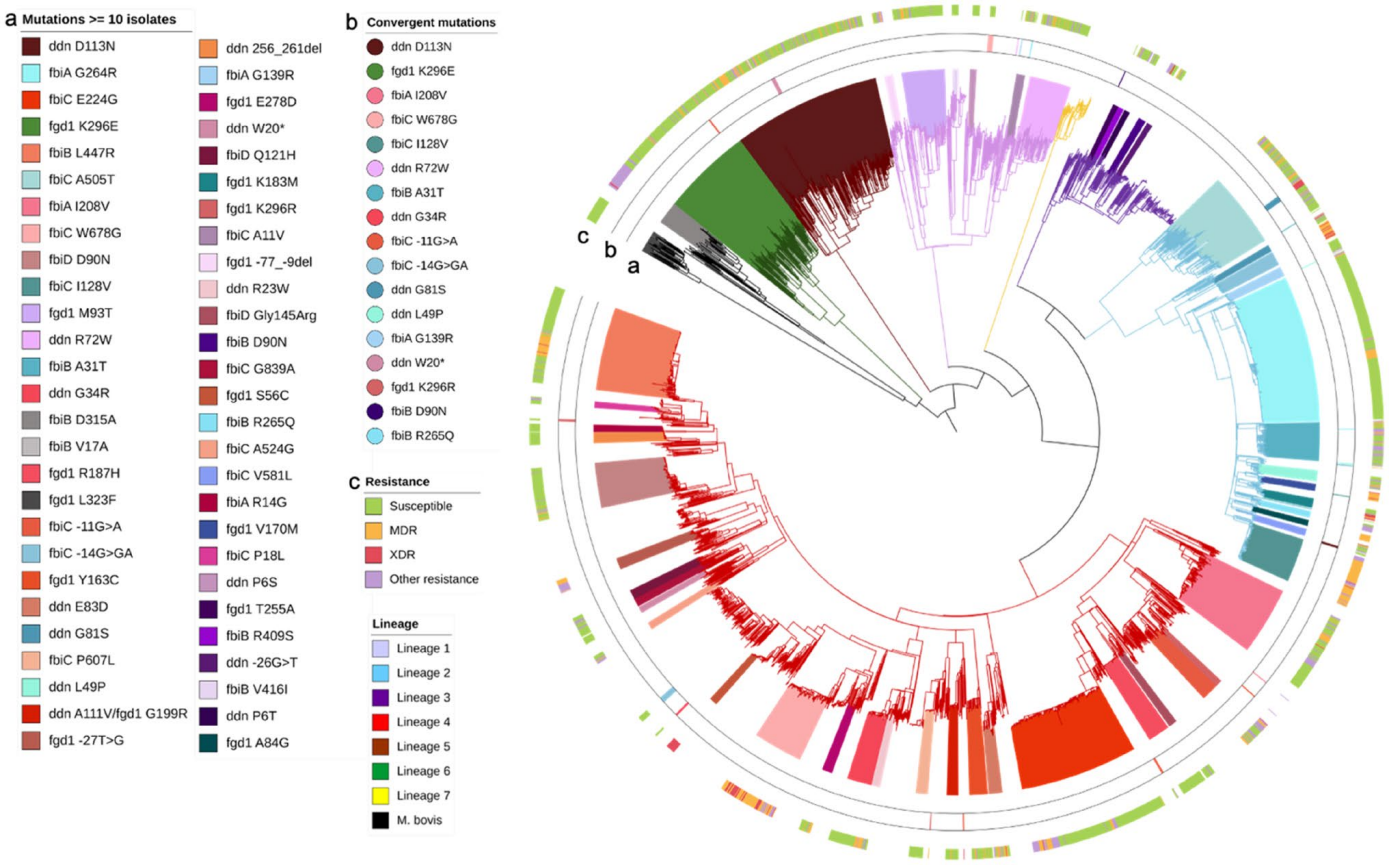
Phylogenetic tree of high frequency mutations (≥ 10 isolates) in delamanid and pretomanid candidate genes (*fgd1* K270M and R64S, *fbiC* −32A > G and T273A, *fbiA* T302M and *fbiB* K448R found in > 290 isolates not represented). Clades formed by isolates harbouring the same mutations are differentiated by colour. The outer (**c**) track shows the resistance phenotype; the second track (**b**) shows the convergent mutations that have arisen in more than one clade; the third track (**a**) shows the clades formed by isolates harbouring the same phylogenetic-related mutations. Branches are coloured by lineage as per legend.

Thirty four of the 117 mutations were found in > 4 isolates and occurred in at least two sub-lineages, appearing up to 4 times in the phylogenetic tree (Table 3, Fig. 3, S6 Table). Eleven of the mutations (*fgd1* K270M, K296E; *ddn* P45L, G81S, G34R, R72W, D113N; *fbiB* D90N, K448R; *fbiC* T273A, W678G) have been identified previously in susceptible samples (S3 Table). The *ddn* L49P mutation, found to be associated with an increment in DLM and PTM MIC^24^, was identified in Beijing strains occurring in genomic clusters from Vietnam, the Netherlands and Mexico, highlighting an ability to disseminate with low fitness impact at an epidemiological level. These isolates were mostly assessed genotypically as non-MDR, and all pre-dated the introduction of DLM as a TB treatment (Table 3). L49 is involved in activation of both DLM and PTM, and L49P is thought to confer cross-resistance to both drugs^24^.

**Table 3:** Mutations in delamanid candidate genes occurring in at least 5 samples and more than one independent clade.

Mutation	Gene	Freq	Sub-lineage (# isolates)	# sub-lineages	Max SNP distance*	# Independent Occurrences	Susc. %	MDR or XDR %	Pre-2014%**	Functional Support***
**K270M**	*fgd1*	3136	4.1.2 + (3135); 2.2.1(1)	3	1329	2	70.1	18.1	84.2	-
**-32A > G**	*fbiC*	639	5, 6, *Bov*(634); 2.2.1(2); 4.3.3(1); 4.2(1); 4.9(1)	7	3264	5	60.1	8.3	63.1	-
**T273A**	*fbiC*	626	4.8(625); 1.1.1(1)	2	330	2	97.9	0.3	93.6	-
**K448R**	*fbiB*	293	3(293)	1	496	3	57.7	30.0	51.1	-
**D113N**	*ddn*	267	5(264); 2.2.1(3)	2	1402	2	70.7	15.4	91.7	-
**K296E**	*fgd1*	162	6(161); 4.1.2.1(1)	2	933	2	87.0	3.7	85.7	-
I208V	*fbiA*	122	4.1.2(121); 4.1.2.1(1)	2	524	2	70.5	11.5	96.9	-
**W678G**	*fbiC*	96	4.3.3(88); 1.1.1(8)	2	87	2	8.3	81.3	90.9	P
**I128V**	*fbiC*	79	2.2.1(79)	0	91	2	0	81.0	100	-
**R72W**	*ddn*	75	1.1.2(75)	1	345	2	76.0	10.7	70.2	S,P
**A31T**	*fbiB*	71	2.2.1(70); 2.2.2(1)	1	238	3	54.9	9.9	100	-
**G34R**	*ddn*	47	4.3.2(44); 4.3.4.2(3)	2	147	2	89.3	8.5	0.0	S,P
**-11G > A**	*fbiC*	37	4.1.2.1(31); 4.1.1.3(3); 6(2); 4.4.2(1)	4	244	4	56.8	16.2	100	-
**-14G > GA**	*fbiC*	34	2.2.1(25); 4.3.4.2.1(9)	2	30	2	26.5	73.5	94.7	-
**G81S**	*ddn*	21	2.2.2(12); 2.1(9)	2	277	2	33.3	52.4	100	S,P
L49P	*ddn*	21	2.2.1.1(21)	1	226	3	57.1	9.5	94.4	S,P
**G139R**	*fbiA*	18	2.2.1(17); 1.1.2(1)	2	30	2	94.4	0	75.0	P
W20*	*ddn*	17	4.5(11); 5(6)	2	241	2	100	0	75.0	-
**K296R**	*fgd1*	16	4.1.2.1(12); 4.8(3); 4.4.1.1(1)	3	75	3	18.8	31.3	37.5	-
**D90N**	*fbiB*	14	3(14)	1	419	2	50.0	14.3	14.3	-
**R265Q**	*fbiB*	13	2.2.1(12); 1.1.2(1)	2	77	3	30.8	0	100	-
**A178T**	*fbiA*	10	1.2.1(8); 4.5(1); 3(1)	3	141	4	70	0	66.7	-
**-43G > A**	*ddn*	9	5(4); 4.2.1(3); 2.2.1(2)	3	244	3	77.8	22.2	100	-
**P131L**	*ddn*	9	4.8(8); 4.3.4.2.1(1)	2	50	2	88.9	0	100	S,P
**G655S**	*fbiC*	9	2.2.1(8); 4.1.2(1)	2	24	2	33.3	0	100	-
**R247W**	*fgd1*	8	3(7); 4.5(1)	2	62	2	100	0	50	P
**V348I**	*fbiB*	8	2.2.2(7); 4.1(1)	2	17	2	100	0	100	-
R304Q	*fbiA*	8	3(8)	2	203	2	87.5	0	50	-
**G325S**	*fbiB*	7	2.2.1(5); 4.9(1); 4.1.2.1(1)	3	63	3	100	0	83.3	-
**P182L**	*fbiB*	6	4.3.4.2.1(3); 6(3)	2	320	2	66.7	16.7	100	-
**M93I**	*fgd1*	6	4.9(3); 4.1.2.1(2); 2.2.1(1)	3	3	3	83.3	0	-	-
**P45L**	*ddn*	5	4.4.1.1(3); 3(1);1.1.1(1)	3	63	3	80	0	100	S,P
**L326F**	*fbiB*	5	4.6.1.1(2); 4.1.2^+^(2); 4.8(1)	4	-	4	100	0	-	-
**T455A**	*fbiC*	5	3(4); 1.1.1(1)	2	236	2	80	0	100	-

Sub-lineages: ^+^ = more than 1 sub-lineage; # = number; * Maximum SNP distance calculated in clades of ≥ 5 isolates; Drug resistance (%): Susc. = Susceptible; ** % of number of samples pre-2014/total number of samples with available collection date; *** Functional support: S = snap2 score ≥ 50; P = Provean Score ≤ −4; M = mCSM predicted stability change (ΔΔG) below − 2; Mutations associated with increased MIC for DLM/PTM in previous studies in bold; mutations associated with susceptibility to DLM/PTM underlined (see S3 Table).

### ddn and fgd1 mutations

Mutations identified in *ddn* included 86 non-synonymous SNPs, 23 small indels, 4 large deletions and 20 mutations in the promoter region. Of these, 10 and 30 have been previously associated with DLM/PTM resistance and susceptibility, respectively (S3 Table). In general, *ddn* amino acid changes were dispersed along the coding region (S8 Figure). Twenty-seven of the (100) novel mutations were indels with 16 causing frameshifts along the coding region (S6 Table; S7 Table; S8 Figure). These indels included 4 large deletions (> 100 bp), identified in low frequencies in MDR-TB isolates (except for 1 susceptible isolate) sourced from China in 2007, before the introduction of DLM as a treatment. Most frameshifts and large deletions were identified in single isolates. Moreover, 6 amino acid changes leading to stop codons and the resultant truncated proteins were identified, including 3 reported (W88*, W27* and Q58*; S3 Table) and 3 unreported variants (W20*, W139*, Y133*). W20* was present in clades consisting of lineage 4.5 (n = 11) and 5 (n = 6) isolates (Fig. 3), where all 16 samples were pan-susceptible. The maximum pairwise SNP difference between lineage 4.5 isolates harbouring *ddn* W20* was 241, suggesting that the variant established itself in that population some time ago. The W88* mutation, which has in vitro evidence of resistance to DLM (S3 Table), appeared within a potential transmission cluster of Beijing MDR-/XDR-TB isolates. Other SNPs known to cause an increment in DLM/PTM MIC (M1T, W88R, Y65S and G53D) were found in 3 or less isolates.

Of the 139 mutations identified in the *fgd1* gene (S9 Figure), six SNPs have been described previously, including two phylogenetically-related (K270M lineage 4.1.2; K296E lineage 6) (S4 Table) with no association with resistance, and two known to increase PTM MIC (G71D and E230K) (see S3 Table). Four frameshifts with disruptive functional consequences for the protein were identified in low frequencies. One isolate was found to harbour K259E, which is a residue involved in F_420_ binding^37^. Of the other mutations, only F79S had a predicted destabilizing effect on the protein (S7 Table).

### fbiA, fbiB, fbiC and fbiD mutations

In total, 119, 136 and 326 mutations were identified in *fbiA*, *fbiB* and *fbiC* respectively (S6 Table; S7 Table). Several mutations that are known to increase DLM/PTM MICs in vitro (S3 Table) were identified (*fbiA* K2E, V154I, I208V, I209V, K250*, S126P, R304Q; *fbiB* P361A; *fbiC* C105R, L228F, L377P, A856P, A835V, S762N), some of them in high frequency, including *fbiA* I208V (n = 122)^36^. Other variants with likely functional impairment of the Fbi proteins comprised one SNP translating into a premature stop codon (*fbiC* G310*) and 12 frameshifts (*fbiA* 2, *fbiB* 1, *fbiC* 9) (S10 Figure; S11 Figure; S12 Figure). In addition, two isolates harboured a 28 amino acid deletion in *fbiA*. One SNP in *fbiA* and 5 SNPs in *fbiC* were found in residues known to be involved in conferring resistance, although different alternate alleles were found compared to those previously reported (S3 Table). Variants previously associated to susceptibility were identified in *fbiA* (Q120R, n = 6; T302M, n = 355), *fbiB (*F220L, n = 2; K448R, n = 293), and *fbiC* (T273A, n = 626; T681I, n = 9) (see S3 Table). Some of these are phylogenetically related (e.g., *fbiA* T302M, *fbiC* T681I). Protein structural modelling revealed predicted deleterious novel mutations in *fbiA* (6), *fbiC* (31), and *fbiB* (4), which may have an impact on the function of their proteins, but not necessarily an association with resistance. For *fbiD*, 66 variants were found, but all are absent in strains from lineages 5, 6 or 7. No deletions or SNPs leading to stop codons were identified in our analysis (S6 Table; S7 Table), including an absence of the 79_80insC indel, which leads to loss of function of the protein and an increase in DLM and PTM MIC values (S3 Table).

### ndh mutations

Three non-synonymous SNPs in *ndh* demonstrated to increase DLM MIC values in *M. smegmatis* (G84V, A175T and M221R)^26^, were not identified in the corresponding residues of our *Mtb* isolates. Five amino acid changes leading to premature stop codon were identified in the data set, and 20 indels leading to frameshifts and 7 large deletions with potential deleterious effects were found. Only the 304_304 deletion was identified in high frequency, namely in 82 MDR-TB isolates from Australia and Papua New Guinea, collected between 2010 and 2015 (S8 Table).

## Discussion

BDQ and DLM are among the last anti-TB drugs approved for the treatment of MDR- and XDR-TB, and have been in use since 2013. Soon after the introduction of BDQ and DLM, resistance to both drugs emerged, and concerns about intrinsic resistance have been raised through the identification of mutations in isolates pre-introduction of both drugs. Similarly, spontaneous resistance-associated variants have been found in BDQ/DLM naïve isolates^15,16,22,38,39^. Recently, PTM has been introduced in combination therapy with BDQ and LNZ for the treatment of XDR-TB cases. A 6-month regimen of PTM, BDQ, and LNZ for XDR-TB or MDR-intolerant TB has been demonstrated to be 90% effective up to 6 months post-treatment, with no event of acquired resistance to PTM^40^. However, the potential for cross-resistance between DLM and PTM exists.

Our study, consisting of > 33,000 isolates, is the largest study to date, and characterised 1,227 variants in nine drug resistance candidate genes for BDQ and DLM. Most mutations (78%), including frameshifts with likely functional effects, were present in isolates collected prior to roll-out of BDQ and DLM. Our analysis has identified phylogenetically related mutations that are unlikely to be drug resistance associated, including in large clades mostly encompassing sensitive profiles to first- and second-line drugs, as well as several mutations that were not considered strain-specific (e.g., *fbiA* G264R, *fbiB* L558R or *fbiC* E224G). As resistance to BDQ and DLM/PTM is relatively rare, newly associated mutations are likely to be discovered through sequencing of resistant isolates in studies of small samples sizes. A potential pitfall of this approach is the spurious association of lineage-defining mutations to drug resistance in candidate genes. An example of this is the G269S mutation in *kasA*, which was initially suggested to cause isoniazid resistance^41^, but in subsequent large studies is associated with T family isolates rather than resistance^42^. To aid researchers in tackling this issue, a list of mutations at high frequency in lineages is provided, and automated detection and annotation of these mutations has now been built into TB-Profiler software^31^. One limitation of our analysis is the relatively low number of sequenced isolates from lineages 5 to 7.

We found mutations known to increase BDQ or DLM MICs in isolates predating the introduction of the three drugs as TB treatments. These included 192_193insG, 193_193del (I67fs) and M146T mutations in *mmpR5* and L49P in *ddn*, with all four variants found in > 20 isolates. Although some studies have observed a correlation between the length of BDQ treatment and the acquisition of mutations in *atpE* or *mmpR5*^43^, the pre-existence of such mutations in BDQ/DLM/PTM naïve isolates has also been described^8,16,22,38,39,44^. The use of CFZ, which is known to cause cross-resistance through mutations in *mmpR5*^8^, has been proposed as a potential explanation. The M146T mutation in *mmpR5* has been identified in a transmission cluster from Eswatini in 2009, where the use of CFZ by some patients could have selected for this variant^35^. Similarly, in Portugal the use of CFZ in the treatment of MDR-/XDR-TB patients may have selected for the *mmpR5* frameshift detected^14^. In the absence of a previous history of CFZ or BDQ use, the treatment of fungal respiratory infections with azoles (i.e., fluconazole or voriconazole) may explain the presence of *mmpR5* mutations^38^. The *mmpR5* 192_193 insertion (I67fs) appears in 10 independent clades, with the largest cluster involving lineage 4 Peruvian samples. High pairwise SNP distances within this clade suggest that this mutation became fixed in this strain pre-2013. The suggested epistatic effect of a *mmpL5* deletion identified in these Peruvian strains^17^ could counteract the potential associated resistance due to I67fs, although there is currently no supporting phenotypic DST data accounting for the 2 mutations (*mmpR5* 192_193ins–*mmpL5* 605_605del). The I67fs frameshift has also been reported in South Africa^16^. A high density of indels were identified along the DNA binding domain of *mmpR5*, which could increase the production of the MmpS5-MmpL5 efflux pump. Fourteen frameshifts were found in the *mmpR5* DNA binding domain, including 2 within the known 192–198 bp hotspot^33^.

For the cross-resistance of DLM and PTM, although both pro-drugs are nitroimidazole derivatives that share the activation pathway, the binding of DLM to Ddn might differ from PTM^24^. However, alteration of specific residues in *ddn*, such as L49P, found in 21 isolates in this study, seemed to confer cross-resistance to both drugs^24^. Nevertheless, as the introduction of PTM in TB treatment regimens is very recent, its use does not provide an explanation for the acquisition of DLM resistance mutations in pre-2014 isolates, but there is evidence of pre-exposure resistance and naturally occurring polymorphisms^44^.

Frameshifts and nonsense non-synonymous mutations are more likely to have a higher functional impact. We have identified several SNPs causing premature stop codons that have already been associated with increments in MIC (*mmpR5* W42*, *ddn* W88* and *fbiA* K250*), as well as others unreported, including one present in eleven lineage 4.5 isolates collected between 2013 and 2015 (*ddn* W20*). Considering the drug susceptibility profile of these isolates and the high SNP distance within the cluster, it seems unlikely that *ddn* W20* emerged from the use of DLM. The *ddn* locus harboured 16 frameshifts mostly in single isolates, likely associated with loss of function. Ddn may have an essential role in recovery from hypoxia, and mutations that keep its native activity would be favoured over those leading to a loss of function^24^.

Protein stability predictions can help to elucidate whether the function of these genes might be altered by non-synonymous SNPs. By using the SUSPECT-BDQ prediction tool, we identified 9 mutations in *atpE* predicted to confer resistance. Among these mutations, E44D was present in a clade of Beijing strains with collection years ranging from 2016 to 2019. However, the sensitive profile of the samples and the monophyletic distribution of the substitution, mean that the acquisition of E44D is unlikely to be a consequence of drug selective pressure, although it could be a naturally occurring polymorphism potentially leading to intrinsic elevated MICs to BDQ for this clade. Moreover, all isolates with the E44D variant also had a SNP in *mmpR5* (D5G) which was predicted not to alter protein stability. Using conservative SNAP2, Provean and mCSM software tools and available crystal structures, we found 51 SNPs with predicted alteration of protein function due to their associated amino acid changes. However, further advanced protein modelling analysis or DST data is required to establish evidence of association with BDQ or DLM/PTM resistance. Similarly, a significant number of SNPs in *mmpR5*, *ddn*, *fgd1*, *fbiA* and *fbiC* were found in residues where amino acid changes leading to increments in MICs have been detected. However, the alternate amino acids identified in this analysis were different. Since differing amino acid changes lead to different values of MIC^24,33^, further investigation is necessary to establish their drug resistance links.

Co-occurrence of mutations in the same gene by isolate was rare. This finding matches previous studies that observed combinations of mutations in *atpE* and *mmpR5* for isolates selected in vitro, whilst clinical isolates tend to harbour unique mutations^38^. For DLM candidate genes, the combination of variants in *fbiC* and *ddn* or *fbiC* and *fgd1* were the most common, potentially due to the greater diversity of these genes, especially *fbiC* and *fgd1*. Since, only one mutation per sample across the nine genes considered was the most prevalent scenario, any additive effects of mutations to reach BDQ and DLM/PTM resistance maybe unlikely. Nevertheless, one limitation of the study is the higher number of samples with a pre-2014 collection date, and therefore the lack of isolates that may have undergone selective pressure under BDQ or DLM/PTM drug regimens. Some of the variants linked to phenotypic drug susceptibility are considered to confer low-level resistance (0.25–0.75 mg/L) or decreases in susceptibility that reach the MIC breakpoint value established by EUCAST (i.e., some frameshifts in *mmpR5*)^15,33^ for MIC determination using the agar proportion method on Middlebrook 7H10/7H11 medium. Noteworthy, evaluation of MIC values by other studies have shown discrepancies between the methods used^33,43,45^. Even assuming that a significant number of these known variants elevate the MICs, some values remain within susceptible ranges, their clinical importance is yet unknown, and they could lead to suboptimal treatment regimens^43^. Moreover, a higher risk of relapse was observed in patients with isolates holding increased MICs but below standard resistance breakpoints for rifampicin and isoniazid^46^. Finally, for *mmpR5*, we observed an elevated risk of mutations among MDR- and XDR-TB isolates, which together with the high proportion of pre-2014 strains, could pose a significant complication for the treatment of BDQ naïve infections.

In summary, we have shown that there are highly frequent resistance-associated variants pre-dating the introduction of BDQ, DLM and PTM, suggesting an intrinsic resistance of these strains, which could constitute a problem for the treatment of MDR-/XDR-TB patients. The use of CFZ and other azoles before the introduction of BDQ could explain the presence of mutations in *mmpR5* in MDR-/XDR-TB isolates. However, the treatment history of some patients is unavailable, including missing sampling dates, making the phylogenetic-based inference of the ages of mutations inaccurate, and the evolutionary pressure by which these mutations have been selected is unclear. Moreover, several frameshifts and nonsense mutations with likely resistance effects have been identified. Since one limitation of the study was the lack of drug susceptibility test data, further investigation is necessary to establish the association between these candidate variants and the phenotypic resistance profiles; ultimately, to elucidate the causative mechanisms of resistance for these new drugs and to achieve better treatment outcomes.

## Methods

Candidate genes for BDQ, DLM and PTM drug resistance were selected based on a review of the literature. Only those genes with experimental evidence of developing mutations under drug exposure either in vitro, in vivo or in *M. tuberculosis* clinical isolates were considered. Specifically, we included 3 genes for BDQ (the target *atpE* and off-targets *mmpR5* (*Rv0678*) and *pepQ*), and 6 genes for DLM/PTM (*ddn*, *fgd1*, *fbiA*, *fbiB*, *fbiC* and *fbiD*) for genetic analysis. Loss of function mutations in the *ndh* gene were considered, as well as in *mmpL5-mmpS5* for epistatic effects with *mmpR5*. Phenotypic drug resistance to CFZ and BDQ was assessed for Portuguese clinical isolates by broth microdilution in Middlebrook 7H9 medium supplemented with oleic acid, albumin, dextrose, catalase (OADC) as per the guidelines of the European Committee on Antimicrobial Susceptibility Testing (EUCAST)^47^. BDQ and CFZ concentrations tested ranged between 4 and 0.016 µg/mL. These Portuguese clinical isolates were retrospectively selected from the Faculty of Pharmacy of the University of Lisbon TB strain bank by screening for isolates with available whole genome sequencing (WGS) data and bearing the *mmpR5* I67fs mutation. Only one isolate met these criteria and four additional *mmpR5* wild-type isolates were included for comparative purposes, including one isolate from the same phylogenetic clade as the mutant isolate (L4.3.4.2/SIT20/LAM1/Lisboa3; SNP distance of 34)^34^. *M. tuberculosis* H37Rv ATCC 27,294 was included as a susceptible reference strain for quality control purpose. Work involving the manipulation of viable *M. tuberculosis* strains and cultures was performed under strict Biosafety Level 3 containment facilities and processed using methods in accordance with the relevant WHO guidelines and institutional regulations.

Publicly available Illumina WGS data for 33,675 *Mtb* isolates spanning 114 countries and all seven main lineages were analysed (see^30^ for raw data accession numbers). Only WGS data with a minimum average coverage of 30, > 90% of reads mapping to H37Rv and > 90% of the genome covered were included. Metadata including collection date and geographical region were incorporated where available. The bioinformatics pipeline for processing raw sequence data is described previously^30^. In brief, raw sequences were aligned with bwa-mem (v0.7.17) software to the H37Rv reference sequence (Genbank accession: NC_000962.3). SNPs and small indels with an allele frequency > 0.95 were identified using GATK HaplotypeCaller (v4.1.4.1). Bcftools csq was used to call amino acid changes. This software handles multiple mutations in the same codon better than alternatives, and in the case *of mmpR5*, some codon numbers differ slightly to previously used nomenclature, and we highlight these (e.g., 193_193del being the same as previously reported I67fs). Large deletions were detected using Delly (v0.8.3, -T DEL) software, and confirmed manually using the IGV (v2.4.9) visualisation tool. TB-Profiler (v3.0) software was used to predict lineage and drug resistance to first and second line drugs^31,48,49^. All high-quality variants identified in the nine candidate genes were extracted. Phylogenetic trees were constructed using concatenated SNP alignments using IQ-Tree (v1.6.12, -m GTR + G + ASC) and visualised together with annotations in iTOL (v5) software. The number of independent acquisitions of variants was calculated by phylogenetic reconstruction followed by ancestral state reconstruction implemented in IQ–Tree (v1.6.12) software.

The R (v3.4.3) statistical package was used to generate the maps. It was also used to perform all statistical analysis, including the fitting of logistic regression models to assess the association of the presence of mutations in candidate genes with the sample collection period, drug resistance status and lineage, where odds ratios and P-values were estimated. The functional effect of SNPs was assessed using SNAP2 and Provean score calculators, and where crystal structures of the *Mtb* proteins were available (PDB: 4NB5, 3R5P, 3B4Y, 4XOM, 6BWG) the mCSM stability predictor was used. For *atpE* SNPs, SUSPECT-BDQ^32^ was used. The protein structures were visualised and annotated using UCSC chimera (https://www.cgl.ucsf.edu/chimera/).

## Supplementary Information

Supplementary Information 1.

Supplementary Information 2.

## Data Availability

Raw sequencing data is available from the ENA short read archive (see^30^ for a list of accession numbers).
