# Supplementary material for: Genetic diversity of candidate loci linked to Mycobacterium tuberculosis resistance to bedaquiline, delamanid and pretomanid
Source: Sci Rep. 2021 Sep 30;11:19431. doi: 10.1038/s41598-021-98862-4 (PMC8484543; doi:10.1038/s41598-021-98862-4)
Supplement: Supplementary file 1 — Supplementary Information 1. [file 41598_2021_98862_MOESM1_ESM.pdf]

**S1 Table**. Geographical region breakdown summary of isolates analysed.

| Region | # countries | # samples | Susc. # (%) | MDR # (%) | XDR # (%) | DR # (%) | Lineages | # pre-2014** |
|---|---|---|---|---|---|---|---|---|
| South Asia | 6 | 941 | 327(34.8) | 456(48.5) | 23(2.4) | 135(14.4) | 1-4 | 305 |
| Europe & Central Asia | 36 | 11323 | 7414(65.5) | 2240(19.8) | 427(3.8) | 1242(11.0) | 1-6 | 3202 |
| Middle East & N. Africa | 9 | 239 | 108(45.2) | 83(34.7) | 23(9.6) | 25(10.5) | 1-4, 6-7 | 149 |
| Sub-Saharan Africa | 34 | 8118 | 6011(74.1) | 1175(14.5) | 259(3.2) | 673(8.3) | 1-4, 6-7 | 5784 |
| Latin America* | 13 | 1463 | 209(14.3) | 923(63.1) | 78(5.3) | 253(17.3) | 1-4 | 800 |
| East Asia & Pacific | 14 | 6068 | 3214(53.0) | 1371(22.6) | 130(2.1) | 1353(22.3) | 1-4, 7 | 3874 |
| North America | 2 | 1962 | 1730(88.2) | 27(1.4) | 0(0) | 205(10.5) | 1-5 | 1658 |
| Unknown | - | 3561 | 2762(77.6) | 228(6.4) | 23(0.7) | 548(15.4) | 1-6 | - |
| **Overall** | **113** | **33675** | **21775(64.7)** | **6503(19.3)** | **963(2.9)** | **4434(13.2)** | **1-7** | **15772** |

* and Caribbean; # = number, Susc. = Susceptible; MDR = multidrug resistant; XDR = extensively drug resistant; DR = Other resistance; ** Number of isolates with date of collection data before 2014.

**S2 Table.** Analysis of the odds of gene mutations.

| Gene | Variable | Odds ratio* | 95% Lower confidence limit | 95% Upper confidence limit | P-value |
|---|---|---|---|---|---|
| ***mmpr5*** | Sensitive | 1.000 | | | |
| | Other DR** | 2.040 | 1.367 | 3.044 | <0.0001 |
| | MDR | 3.781 | 2.765 | 5.171 | <0.0001 |
| | XDR | 9.937 | 6.626 | 14.904 | <0.0001 |
| ***ddn*** | Sensitive | 1.000 | | | |
| | Other DR** | 1.019 | 0.684 | 1.517 | 0.926 |
| | MDR | 1.559 | 1.104 | 2.202 | 0.012 |
| | XDR | 2.268 | 1.150 | 4.474 | 0.018 |

* adjusted for lineage and year of collection; ** non-MDR; MDR multi-drug resistant; XDR extensively drug resistant

**S4 Table.** Phylogenetic mutations with >50% of allele frequency within a sub-lineage.

| Mutation | Gene | Freq | Sub-lineage (# isolates) | Freq (%) in sub-lineage | #sub-lin. | Max. SNP dist. | # Indep. Occur. | Susc. % | MDR /XDR % | Pre-2014 % * |
|---|---|---|---|---|---|---|---|---|---|---|
| K270M | *fgd1* | 3136 | 4.1.2[+](3135); 2.2.1(1) | 96.0 | 3 | 1329 | 2 | 70.1 | 18.1 | 84.2 |
| -32A>G | *fbiC* | 639 | 5,6,*Bov*(634); 2.2.1(2); 4.3.3(1); 4.2(1); 4.9(1) | 98.4 | 7 | 3264 | 5 | 60.1 | 8.3 | 63.1 |
| R64S | *fgd1* | 471 | 1.1.1[+](471) | 54.4 | 2 | 515 | 1 | 77.9 | 2.1 | 99.1 |
| T302M | *fbiA* | 355 | 4.1.1.1(355) | 99.7 | 1 | 337 | 1 | 82.8 | 9.9 | 84.8 |
| D113N | *ddn* | 267 | 5(264); 2.2.1(3) | 100 | 2 | 1402 | 2 | 70.7 | 15.4 | 91.7 |
| K296E | *fgd1* | 162 | 6(161); 4.1.2.1(1) | 98.2 | 2 | 933 | 2 | 87.0 | 3.7 | 85.7 |
| A505T | *fbiC* | 135 | 2.1(135) | 100 | 1 | 486 | 1 | 61.5 | 20.0 | 95.1 |
| P69L | *pepQ* | 141 | 4.4.1.2(141) | 100 | 1 | 284 | 1 | 89.4 | 1.4 | 92.1 |

Drug resistance (%): Susc. = Susceptible; * % of number of samples pre-2014/total number of samples with available collection date; mutations associated with no significant change in minimum inhibitory concentration are underlined (with MIC usually <0.06 mg/L for BDQ and <0.2 mg/L for DLM/PTM; see **S3 Table**); Bedaquiline (BDQ), delamanid (DLM); pretomanid (PTM).

**S5 Table.** All mutations (in >1 isolate) in bedaquiline (BDQ) candidate genes found in the 33k isolates

| Mutation | Gene | Freq | Sub-lineage(# isolates) | # sub-lin. | # Indep. Occur. | Susc. % | MDR /XDR % | Pre-2014 % * | Functional Support ** |
|---|---|---|---|---|---|---|---|---|---|
| P69L | *pepQ* | 141 | 4.4.1.2(141) | 1 | 1 | 89.4 | 1.4 | 92.1 | P |
| -11C>A | *mmpR5* | 124 | 2.2.1(122); 4.3.2.1(1); 1.1.1(1) | 3 | 3 | 12.1 | 76.6 | 93.1 | - |
| **192_193insG (I67fs)** | *mmpR5* | 44 | 4(34); 2.2.1(4); 3(2); 4.9(1); 4.8(1); 4.5(1); 1.1.1(1) | 7 | 10 | 0 | 86.4 | 100 | - |
| G197R | *pepQ* | 38 | 4.3.4.1(37); 2.2.1(1) | 2 | 2 | 52.6 | 47.4 | 72.2 | S,P |
| R7Q | *pepQ* | 35 | 3(35) | 1 | 1 | 68.6 | 22.9 | 66.7 | - |
| T354A | *pepQ* | 27 | 3(27) | 1 | 1 | 100 | 0 | 0 | - |
| K94N | *pepQ* | 23 | 3.1.1(22); 4.1.2(1) | 2 | 2 | 95.7 | 0 | 100 | - |
| **M146T** | *mmpR5* | 21 | 4.4.1.1(20); 2.2.2(1) | 2 | 2 | 0 | 100 | - | S,M |
| D5G | *mmpR5* | 18 | 2.2.1(17); 4.1.2.1(1) | 2 | 2 | 94.4 | 0 | 75.0 | - |
| E44D | *atpE* | 17 | 2.2.1(17) | 1 | 1 | 94.1 | 0 | 75.0 | B,S |
| A242T | *pepQ* | 17 | 2.2.1.1(17) | 1 | 1 | 58.8 | 5.9 | 100 | - |
| **193_193del (I67fs)** | *mmpR5* | 16 | 4.3.4.2(10); 2.2.1(3); 4.7(2); 4.3.3.1(1) | 4 | 5 | 0 | 100 | 83.3 | - |
| D20G | *pepQ* | 15 | 4.6(15) | 1 | 1 | 100 | 0 | 20.0 | P |
| **141_142insC** | *mmpR5* | 15 | 2.2$^{+}$(8); 4.1.2$^{+}$(2); 4.3$^{+}$(2); 4.4.1.1(1); 3(2) | 8 | 11 | 6.7 | 86.7 | 85.7 | - |
| -49T>C | *mmpR5* | 12 | 3.1.2.1(12) | 1 | 1 | 75.0 | 8.3 | 0 | - |
| G87R | *mmpR5* | 11 | 1.1.2(11) | 1 | 1 | 100 | 0 | 80.0 | S,P |
| V20A | *mmpR5* | 10 | 4.1.2.1(8); 4.3.2.1(1); 2.2.1(1) | 3 | 3 | 90.0 | 10.0 | 83.3 | M |
| **L117R** | *mmpR5* | 9 | 3(5); 4.3.4.2(2); 4.2.2(1); 4.1(1) | 4 | 5 | 44.4 | 44.4 | 100 | S |
| N4T | *mmpR5* | 9 | 3(9) | 1 | 1 | 55.5 | 33.3 | - | - |
| V3I | *mmpR5* | 9 | 4.3.4.2(9) | 1 | 1 | 22.2 | 66.7 | - | - |
| V211A | *pepQ* | 9 | 3.1.1(9) | 1 | 1 | 100 | 0 | 100 | - |
| E115A | *pepQ* | 8 | 4.1.2.1(8) | 1 | 1 | 100 | 0 | 0 | - |
| L32S | *mmpR5* | 8 | 2.2.1(8) | 1 | 3 | 0 | 87.5 | 50.0 | S,M |
| **138_139insG** | *mmpR5* | 7 | 2.2.1(7) | 1 | 1 | 0 | 100 | 100 | - |
| D141H | *mmpR5* | 7 | 2.2.1(6); 1.1.3(1) | 2 | 2 | 14.3 | 57.1 | 100 | S,P |
| **R90C** | *mmpR5* | 7 | 2.2.1(6); 4.1.1.3(1) | 2 | 4 | 85.7 | 0 | 50.0 | - |
| **418_419insG** | *mmpR5* | 7 | 4.1.2.1(7) | 1 | 1 | 0 | 0 | - | - |
| **G121R** | *mmpR5* | 7 | 2.2.2(5); 3(1); 4.4.1.1(1) | 3 | 3 | 0 | 100 | 100 | S,P |
| T341A | *pepQ* | 6 | 2.1(6) | 1 | 1 | 50 | 33.3 | 100 | P |
| D119E | *mmpR5* | 6 | 4.9(6) | 1 | 1 | 100 | 0 | 0 | - |
| S2R | *mmpR5* | 6 | 3(6) | 1 | 1 | 33.3 | 66.7 | - | - |
| G126D | *mmpR5* | 5 | 1.2.1(5) | 1 | 1 | 100 | 0 | 50 | - |
| V298I | *pepQ* | 5 | 4.8(5) | 1 | 1 | 100 | 0 | 100 | - |

| | | | | | | | | | |
|---|---|---|---|---|---|---|---|---|---|
| A153G | *pepQ* | 5 | 4.7(5) | 1 | 1 | 100 | 0 | - | - |
| **N98D** | *mmpR5* | 5 | 4.1.2.1(2); 4.4.1.1(2); 2.2.1(1) | 3 | 3 | 0 | 80.0 | 100 | - |
| V85G | *mmpR5* | 5 | 2.2.1(5) | 1 | 1 | 0 | 100 | 100 | - |
| T363I | *pepQ* | 5 | 4.8(5) | 1 | 1 | 100 | 0 | 100 | S,P |
| E55D | *mmpR5* | 4 | 2.2.1(4) | 1 | 1 | 75.0 | 25.0 | 100 | - |
| G162E | *mmpR5* | 4 | 1.1.2(4) | 1 | 1 | 100 | 0 | 100 | - |
| S99R | *pepQ* | 4 | 4.2.1(4) | 1 | 1 | 50.0 | 0 | - | - |
| V149I | *mmpR5* | 4 | 1.1.2(4) | 1 | 1 | 0 | 0 | - | - |
| A224V | *pepQ* | 4 | 5(2); 2.2.1.1(1); 1.2.2(1) | 3 | 3 | 75.0 | 25.0 | 50.0 | - |
| D26A | *pepQ* | 4 | 4.3.3(4) | 1 | 1 | 0 | 100 | 100 | P |
| A196V | *pepQ* | 4 | 2.2.1(4) | 1 | 2 | 25.0 | 75.0 | 100 | - |
| I193T | *pepQ* | 4 | 3(4) | 1 | 1 | 25.0 | 25.0 | - | S,P |
| N148H | *mmpR5* | 4 | 1.1.2(4) | 1 | 1 | 100 | 0 | 75.0 | - |
| V39I | *atpE* | 4 | 4.3.3(4) | 1 | 1 | 100 | 0 | 0 | - |
| -29G>A | *mmpR5* | 3 | 3(2); 1.2.2(1) | 2 | 2 | 100 | 0 | 66.7 | - |
| R109W | *mmpR5* | 3 | 3(2); 1.2.2(1) | 2 | 2 | 66.7 | 33.3 | 100 | - |
| M111T | *mmpR5* | 3 | 1.1.2(3) | 1 | 1 | 100 | 0 | - | M |
| T341I | *pepQ* | 3 | 3(2); 1.1.2(1) | 2 | 2 | 100 | 0 | - | P |
| -37T>C | *mmpR5* | 3 | 4.3.4.1(3) | 1 | 1 | 100 | 0 | 100 | - |
| G41A | *mmpR5* | 3 | 4.3.2(3) | 1 | 1 | 33.3 | 0 | - | - |
| G41C | *pepQ* | 3 | 4.7(3) | 1 | 1 | 100 | 0 | - | - |
| A243V | *pepQ* | 3 | 5(3) | 1 | 1 | 100 | 0 | 0 | - |
| G126S | *mmpR5* | 3 | 1.2.1(3) | 1 | 1 | 0 | 100 | 0 | - |
| -3C>CT | *mmpR5* | 3 | 4.3.3(3) | 1 | 1 | 0 | 100 | 100 | - |
| **S53L** | *mmpR5* | 3 | 4.1.1.3(2); 2.2.1(1) | 2 | 2 | 66.7 | 0 | - | - |
| V120M | *mmpR5* | 3 | 4.1.1(1); 1.1.2(1); 1.2.1(1) | 3 | 3 | 66.7 | 0 | 50 | - |
| **16_16del** | *mmpR5* | 3 | 2.2.2(1); 2.2.1+(2) | 3 | 3 | 33.3 | 66.7 | 100 | - |
| T56I | *pepQ* | 3 | 4.5(2); 2.2.1(1) | 2 | 2 | 33.3 | 66.7 | 100 | P |
| -30CG>C | *mmpR5* | 3 | 4.5(3) | 1 | 1 | 0 | 100 | 100 | - |
| -21T>C | *mmpR5* | 3 | 4.5(3) | 1 | 1 | 0 | 100 | 100 | - |
| S63N | *mmpR5* | 3 | 4.4.2(2) | 1 | 1 | 0 | 100 | 100 | S |
| -31GGCTACCAGA>G | *atpE* | 3 | 4.4.2(3) | 1 | 1 | 0 | 0 | 100 | - |
| A59T | *mmpR5* | 3 | 2.2.1(3) | 1 | 1 | 0 | 100 | 100 | - |
| -38ATACCGAACG>A | *mmpR5* | 3 | 1.1(3) | 1 | 1 | 66.7 | 0 | - | - |
| L163V | *pepQ* | 3 | 1.1.1(3) | 1 | 1 | 100 | 0 | - | - |
| T2K | *pepQ* | 3 | 4.2.2(3) | 1 | 1 | 66.7 | 0 | - | - |
| A128V | *mmpR5* | 3 | 1.2.2(3) | 1 | 1 | 100 | 0 | 0 | - |
| D283G | *pepQ* | 3 | 4.1.2.1(3) | 1 | 1 | 100 | 0 | 33.3 | - |
| D26G | *pepQ* | 3 | 3(2); 2.2.1(1) | 2 | 3 | 33.3 | 33.3 | 100 | P |

| | | | | | | | | | |
|---|---|---|---|---|---|---|---|---|---|
| V343L | *pepQ* | 3 | 1.2.2(3) | 1 | 1 | 100 | 0 | 100 | - |
| M23V | *mmpR5* | 3 | 2.2.1(3) | 1 | 1 | 0 | 0 | 100 | - |
| -9G>C | *mmpR5* | 3 | 4.1.2(3) | 1 | 1 | 100 | 0 | 100 | - |
| P97L | *mmpR5* | 2 | 4.1(1); 3(1) | 2 | 2 | 100 | 0 | - | P |
| T78I | *atpE* | 2 | 3(2) | 1 | 1 | 100 | 0 | - | B |
| V80A | *atpE* | 2 | 3(2) | 1 | 1 | 100 | 0 | - | B |
| **M139T** | *mmpR5* | 2 | 4.5(1); 2.2.1(1) | 2 | 2 | 50.0 | 50.0 | 100 | M |
| A84V | *mmpR5* | 2 | 4.3.4.2.1(1); 2.2.1(1) | 2 | 2 | 50.0 | 50.0 | - | - |
| Y145H | *mmpR5* | 2 | 3(2) | 1 | 1 | 0 | 100 | - | S,M |
| -41C>G | *mmpR5* | 2 | 4.1.2.1(2) | 1 | 1 | 100 | 0 | - | - |
| V85I | *mmpR5* | 2 | 4.8(2) | 1 | 1 | 100 | 0 | - | - |
| P129S | *mmpR5* | 2 | 4.8(1); 2.1(1) | 2 | 2 | 50.0 | 0 | 100 | - |
| V158L | *pepQ* | 2 | 4.4.1.1(2) | 1 | 1 | 100 | 0 | 100 | - |
| T91I | *mmpR5* | 2 | 5(2) | 1 | 1 | 0 | 100 | - | - |
| L74M | *mmpR5* | 2 | 6(1); 4.2.2.1(1) | 2 | 2 | 0 | 100 | - | - |
| Y92C | *mmpR5* | 2 | 4.3.3(1); 4.4.2(1) | 2 | 2 | 0 | 100 | 100 | P,M |
| F93L | *mmpR5* | 2 | 4.4.1.1(1); 4.2.2(1) | 2 | 2 | 50.0 | 50.0 | 100 | S,P |
| Y229C | *pepQ* | 2 | 4.8(2) | 1 | 1 | 100 | 0 | - | S,P |
| R105G | *mmpR5* | 2 | 1.2.1(1); 2.2.1(1) | 2 | 2 | 0 | 50.0 | 100 | - |
| -41T>C | *atpE* | 2 | 1.1.2(1); *Bov*(1) | 2 | 2 | 50.0 | 0 | 0 | - |
| R156* | *mmpR5* | 2 | 5(1); .4.2(1) | 2 | 2 | 0 | 100 | 100 | - |
| R50Q | *mmpR5* | 2 | 2.2.2(2) | 1 | 1 | 0 | 100 | 0 | - |
| E54A | *mmpR5* | 2 | 1.1.1(1); 4.1.2.1(1) | 2 | 2 | 50.0 | 50.0 | 100 | - |
| V214F | *pepQ* | 2 | 4.2.1(2) | 1 | 1 | 100 | 0 | 100 | - |
| R109L | *mmpR5* | 2 | 3(2) | 1 | 1 | 100 | 0 | 100 | - |
| V101A | *pepQ* | 2 | 4.4.2(2) | 1 | 1 | 0 | 0 | 100 | - |
| R30S | *mmpR5* | 2 | 4.5(2) | 1 | 1 | 100 | 0 | 100 | M |
| Q22E | *mmpR5* | 2 | 2.2.1(2) | 1 | 1 | 0 | 100 | 100 | - |
| L44P | *mmpR5* | 2 | 2.2.1(2) | 1 | 1 | 0 | 100 | 100 | S,P |
| -7G>GA | *mmpR5* | 2 | 2.2.1.1(2) | 1 | 1 | 0 | 100 | 100 | - |
| **136_137insG** | *mmpR5* | 2 | 2.2.1(2) | 1 | 1 | 0 | 0 | 100 | - |
| V101L | *pepQ* | 2 | 4.5(2) | 1 | 1 | 100 | 0 | 100 | - |
| R206Q | *pepQ* | 2 | 1.1.1(2) | 1 | 1 | 100 | 0 | - | - |
| P366T | *pepQ* | 2 | 1.1.1(2) | 1 | 1 | 100 | 0 | - | - |
| P359L | *pepQ* | 2 | 1.1.1(1); 6(1) | 2 | 2 | 50.0 | 50.0 | 100 | - |
| A12T | *pepQ* | 2 | 1.2.2(2) | 1 | 1 | 100 | 0 | - | - |
| A90V | *pepQ* | 2 | 4.1.1.1(2) | 1 | 1 | 100 | 0 | - | - |
| R96G | *mmpR5* | 2 | 4.2.1(2) | 1 | 1 | 100 | 0 | 0 | S |
| F27V | *mmpR5* | 2 | 4.3.3(2) | 1 | 1 | 100 | 0 | - | M |
| **V85A** | *mmpR5* | 2 | 1.2.2(2) | 1 | 1 | 0 | 0 | 0 | - |
| D165N | *mmpR5* | 2 | 3(2) | 1 | 1 | 0 | 50.0 | - | - |
| **A153P** | *mmpR5* | 2 | 2.2.1(2) | 1 | 1 | 0 | 100 | - | S |
| M17V | *mmpR5* | 2 | 4.9(2) | 1 | 1 | 0 | 100 | - | - |

| | | | | | | | | | |
|---|---|---|---|---|---|---|---|---|---|
| G116V | *pepQ* | 2 | 4.1.1.3(2) | 1 | 1 | 0 | 100 | - | - |
| 128_137del | *mmpR5* | 2 | 4.1.1.3(1); 4.1.2.1(1) | 2 | 2 | 0 | 100 | - | - |
| A124V | *pepQ* | 2 | 4.1.1.3(1); 1.1.1(1) | 2 | 2 | 50.0 | 50.0 | 100 | - |
| L117P | *mmpR5* | 2 | 2.2.1(2) | 1 | 1 | 0 | 100 | 100 | S,P |
| **V1A** | *mmpR5* | 2 | 2.2.1(1); 4.2.2(1) | 2 | 2 | 50.0 | 50.0 | 100 | - |
| K241T | *pepQ* | 2 | 4.1.2.1(2) | 1 | 1 | 100 | 0 | 100 | - |
| **465_466insC** | *mmpR5* | 2 | 4.1.2.1(2) | 1 | 1 | 0 | 100 | - | - |
| **274_275insA** | *mmpR5* | 2 | 4.3.4.2.1(1); 2.2.1(1) | 2 | 2 | 50.0 | 50.0 | 100 | - |
| 778866_779 429del | *mmpR5* | 2 | 4.3.4.2.1(2) | 1 | 1 | 100 | 0 | 100 | - |
| G58S | *pepQ* | 2 | 4.3.4.2.1(2) | 1 | 1 | 100 | 0 | 100 | P |
| I220L | *pepQ* | 2 | 4.3.4.2(2) | 1 | 1 | 0 | 100 | 0 | - |
| D151G | *pepQ* | 2 | 4.4(2) | 1 | 1 | 0 | 100 | 100 | P |
| Q22R | *mmpR5* | 2 | 4.4.2(2) | 1 | 1 | 0 | 0 | 100 | - |

Sub-lineages: $^{+}$ = more than 1 sub-lineage; # = number; Drug resistance (%): Susc. = Susceptible; * % of number of samples pre-2014/total number of samples with available collection date; ** Functional support: S = snap2 score >= 50; P = Provean Score =< -4; M = mCSM predicted stability change (ΔΔG) below -2; B = Predicted as resistant by SUSPECT-BDQ (only available for *atpE*). Mutations associated with increased minimum inhibitory concentration (MIC) for bedaquiline (BDQ) in previous studies in bold; mutations associated with susceptibility to BDQ underlined (see **S3 Table**).

**S6 Table.** All mutations (seen >1 samples) in Delamanid (DLM) /Pretomanid (PTM) candidate genes found in the 33k isolates.

| Mutation | Gene | Freq | Sub-lineage(# isolates) | # sub-lin. | # Indep Occur. | Susc. % | MDR/XDR % | Pre-2014 % * | Functional Support ** |
|---|---|---|---|---|---|---|---|---|---|
| K270M | *fgd1* | 3136 | 4.1.2$^{+}$(3135); 2.2.1(1) | 3 | 2 | 70.1 | 18.1 | 84.2 | - |
| -32A>G | *fbiC* | 639 | 5, 6, *Bov*(634); 2.2.1(2); 4.3.3(1); 4.2(1); 4.9(1) | 7 | 5 | 60.1 | 8.3 | 63.1 | - |
| T273A | *fbiC* | 626 | 4.8(625); 1.1.1(1) | 2 | 2 | 97.9 | 0.3 | 93.6 | - |
| R64S | *fgd1* | 471 | 1.1.1$^{+}$(471) | 2 | 1 | 77.9 | 2.1 | 99.1 | - |
| T302M | *fbiA* | 355 | 4.1.1.1(355) | 1 | 1 | 82.8 | 9.9 | 84.8 | - |
| K448R | *fbiB* | 293 | 3(293) | 1 | 3 | 57.7 | 30 | 51.1 | - |
| D113N | *ddn* | 267 | 5(264); 2.2.1(3) | 2 | 2 | 70.7 | 15.4 | 91.7 | - |
| G264R | *fbiA* | 261 | 2.2.1(261) | 1 | 1 | 91.9 | 6.1 | 100 | P |
| E224G | *fbiC* | 210 | 4.1.1.3(210) | 1 | 1 | 74.8 | 10 | 80 | S |
| K296E | *fgd1* | 162 | 6(161); 4.1.2.1(1) | 2 | 2 | 87 | 3.7 | 85.7 | - |
| L447R | *fbiB* | 148 | 4.8(148) | 1 | 1 | 73.6 | 23 | 95.9 | - |
| A505T | *fbiC* | 135 | 2.1(135) | 1 | 1 | 61.5 | 20 | 95.1 | - |
| **I208V** | *fbiA* | 122 | 4.1.2(121); 4.1.2.1(1) | 2 | 2 | 70.5 | 11.5 | 96.9 | - |
| W678G | *fbiC* | 96 | 4.3.3(88); 1.1.1(8) | 2 | 2 | 8.3 | 81.2 | 90.9 | P |
| D90N | *fbiD* | 80 | 4.9(80) | 1 | 1 | 87.5 | 6.25 | 100 | - |
| I128V | *fbiC* | 79 | 2.2.1(79) | 1 | 2 | 0 | 81 | 100 | - |
| M93T | *fgd1* | 76 | 1.2.2(76) | 1 | 1 | 85.5 | 9.2 | 100 | - |
| R72W | *ddn* | 75 | 1.1.2(75) | 1 | 2 | 76 | 10.7 | 70.2 | S,P |
| A31T | *fbiB* | 71 | 2.2.1(70); 2.2.2(1) | 1 | 3 | 54.9 | 9.9 | 100 | - |
| G34R | *ddn* | 47 | 4.3.2(44); 4.3.4.2(3) | 2 | 2 | 89.3 | 8.5 | 0 | S,P |
| D315A | *fbiB* | 40 | *Bov*(40) | 1 | 1 | 0 | 5 | 0 | - |
| V17A | *fbiB* | 39 | *Bov*(39) | 1 | 1 | 100 | 0 | 0 | - |
| R187H | *fgd1* | 39 | 4.1.1.1(39) | 1 | 1 | 100 | 0 | 100 | - |
| L323F | *fgd1* | 38 | *Bov*(38) | 1 | 1 | 100 | 0 | 0 | - |
| -11G>A | *fbiC* | 37 | 4.1.2.1(31); 4.1.1.3(3); 6(2); 4.4.2(1) | 4 | 4 | 56.8 | 16.2 | 100 | - |
| -14G>GA | *fbiC* | 34 | 2.2.1(25); 4.3.4.2.1(9) | 2 | 2 | 26.5 | 73.5 | 94.7 | - |
| Y163C | *fgd1* | 32 | 4(32) | 1 | 1 | 81.3 | 15.6 | 28.6 | P |
| E83D | *ddn* | 24 | 4.2.1(24) | 1 | 1 | 33.3 | 45.9 | 100 | |
| G81S | *ddn* | 21 | 2.2.2(12); 2.1(9) | 2 | 2 | 33.3 | 52.4 | 100 | S,P |

| | | | | | | | | | |
|---|---|---|---|---|---|---|---|---|---|
| P607L | *fbiC* | 21 | 4.4.1.1(21) | 1 | 1 | 100 | 0 | 100 | P |
| **L49P** | *ddn* | 21 | 2.2.1.1(21) | 1 | 3 | 57.1 | 9.5 | 94.4 | S,P |
| A111V | *ddn* | 20 | 4.4.2(20) | 1 | 1 | 90 | 10 | 100 | S,P |
| G199R | *fgd1* | 20 | 4.4.2(20) | 1 | 1 | 90 | 10 | 100 | P |
| -27T>G | *fgd1* | 20 | 4.6(20) | 1 | 1 | 0 | 40 | 0 | - |
| 256_261del | *ddn* | 19 | 4.8(19) | 1 | 1 | 100 | 0 | 0 | - |
| G139R | *fbiA* | 18 | 2.2.1(17); 1.1.2(1) | 2 | 2 | 94.4 | 0 | 75 | P |
| E278D | *fgd1* | 18 | 4.3.3(18) | 1 | 1 | 66.7 | 27.8 | 66.7 | - |
| W20* | *ddn* | 17 | 4.5(11); 5(6) | 2 | 2 | 100 | 0 | 75 | - |
| Q121H | *fbiD* | 17 | 4.5(17) | 1 | 1 | 76.5 | 0 | 100 | - |
| K183M | *fgd1* | 16 | 2.2.1(16) | 1 | 1 | 43.8 | 50 | 100 | - |
| K296R | *fgd1* | 16 | 4.1.2.1(12); 4.8(3); 4.4.1.1(1) | 3 | 3 | 18.8 | 31.3 | 37.5 | - |
| A11V | *fbiC* | 15 | 1.1.2(15) | 1 | 1 | 66.7 | 13.3 | 53.8 | - |
| -77_-9del | *fgd1* | 15 | 1.2.1(15) | 1 | 1 | 40 | 20 | 100 | - |
| R23W | *ddn* | 15 | 4.3.2(15) | 1 | 1 | 33.3 | 46.7 | 0 | S,P |
| G145R | *fbiD* | 15 | 4.1.1.1(15) | 1 | 1 | 86.7 | 0 | 80 | S,P |
| D90N | *fbiB* | 14 | 3(14) | 1 | 2 | 50 | 14.3 | 14.3 | - |
| G839A | *fbiC* | 14 | 4.5(14) | 1 | 1 | 85.7 | 0 | 85.7 | - |
| S56C | *fgd1* | 14 | 4.3.4.2(14) | 1 | 1 | 0 | 100 | 0 | - |
| R265Q | *fbiB* | 13 | 2.2.1(12); 1.1.2(1) | 2 | 3 | 30.8 | 0 | 100 | - |
| A524G | *fbiC* | 13 | 4(13) | 1 | 1 | 53.8 | 7.7 | 100 | - |
| V581L | *fbiC* | 12 | 2.2.1(12) | 1 | 1 | 58.3 | 16.6 | 100 | - |
| R14G | *fbiA* | 12 | 4.8(12) | 1 | 1 | 100 | 0 | 100 | S,P |
| V170M | *fgd1* | 12 | 2.2.1(12) | 1 | 1 | 0 | 100 | 33.3 | - |
| P18L | *fbiC* | 11 | 4.8(11) | 1 | 1 | 72.7 | 0 | 100 | - |
| P6S | *ddn* | 11 | 1.1.1(11) | 1 | 1 | 100 | 0 | 100 | - |
| T255A | *fgd1* | 10 | 3(10) | 1 | 1 | 60 | 10 | 0 | - |
| R409S | *fbiB* | 10 | 3(10) | 1 | 1 | 80 | 0 | 100 | P |
| -26G>T | *ddn* | 10 | 3(10) | 1 | 1 | 70 | 20 | 100 | - |
| V416I | *fbiB* | 10 | 1.1.3(10) | 1 | 1 | 100 | 0 | 100 | - |
| P6T | *ddn* | 10 | 3(10) | 1 | 1 | 80 | 0 | 0 | P |
| A178T | *fbiA* | 10 | 1.2.1(8); 4.5(1); 3(1) | 2 | 4 | 70 | 0 | 66.7 | - |
| A84G | *fgd1* | 10 | 2.2.1(10) | 1 | 1 | 0 | 100 | 100 | - |
| -43G>A | *ddn* | 9 | 5(4); 4.2.1(3); 2.2.1(2) | 3 | 3 | 30.8 | 0 | 100 | - |
| A199T | *fbiA* | 9 | 2.2.2(9) | 1 | 1 | 0 | 88.9 | 100 | - |
| R230Q | *fbiB* | 9 | 1.1.3(9) | 1 | 1 | 100 | 0 | - | - |
| 2546_2547insCACATACGCCCTGCTTGCG | *fbiC* | 9 | 4.6(9) | 1 | 1 | 77.8 | 0 | 40 | - |
| W589R | *fbiC* | 9 | 4.3.4.2(9) | 1 | 1 | 100 | 0 | - | - |
| R30S | *ddn* | 9 | 2.2.1(9) | 1 | 1 | 11.1 | 55.6 | 100 | S,P |
| P131L | *ddn* | 9 | 4.8(8); 4.3.4.2.1(1) | 2 | 2 | 88.9 | 0 | 100 | S,P |

| | | | | | | | | | |
|---|---|---|---|---|---|---|---|---|---|
| T681I | *fbiC* | 9 | 2.2.1(9) | 1 | 1 | 77.8 | 11.1 | 100 | P |
| 363_386del | *ddn* | 9 | 4.5(9) | 1 | 1 | 77.8 | 11.1 | - | - |
| A143V | *fbiC* | 9 | 4.3.4.2.1(9) | 1 | 1 | 100 | 0 | 100 | - |
| G655S | *fbiC* | 9 | 2.2.1(8); 4.1.2(1) | 2 | 2 | 33.3 | 0 | 100 | - |
| I13L | *fbiC* | 9 | *Bov*(9) | 1 | 1 | 0 | 11.1 | 0 | - |
| A197V | *fbiD* | 9 | 1.1.3(9) | 1 | 1 | 100 | 0 | - | - |
| G572C | *fbiC* | 8 | 2.2.1(8) | 1 | 1 | 0 | 75 | 100 | - |
| R247W | *fgd1* | 8 | 4.5(1); 3(7) | 2 | 2 | 100 | 0 | 50 | P |
| V348I | *fbiB* | 8 | 4.1(1); 2.2.2(7) | 2 | 2 | 100 | 0 | 100 | - |
| **R304Q** | *fbiA* | 8 | 3(8) | 1 | 2 | 87.5 | 0 | 50 | - |
| -24C>A | *ddn* | 7 | 4.1.1.2(7) | 1 | 1 | 85.7 | 0 | 100 | - |
| T687M | *fbiC* | 7 | 1.1.2(7) | 1 | 1 | 42.9 | 42.9 | - | - |
| A349V | *fbiC* | 7 | 1.1.1(7) | 1 | 1 | 85.7 | 0 | 100 | S |
| A345G | *fbiC* | 7 | 4.3.4.2.1(7) | 1 | 1 | 85.7 | 0 | 100 | - |
| A2V | *fgd1* | 7 | 4.2.2(7) | 1 | 1 | 100 | 0 | 0 | - |
| **S762N** | *fbiC* | 7 | 3(7) | 1 | 1 | 42.8 | 28.6 | 0 | - |
| D312G | *fbiA* | 7 | 4.8(7) | 1 | 1 | 71.4 | 14.3 | 100 | P |
| -13A>G | *fbiC* | 7 | 2.2.1(7) | 1 | 1 | 85.7 | 14.3 | 100 | - |
| V188F | *fbiA* | 7 | 1.2.1(7) | 1 | 1 | 28.6 | 14.3 | 33.3 | P |
| G325S | *fbiB* | 7 | 4.9(1); 4.1.2.1(1); 2.2.1(5) | 3 | 3 | 100 | 0 | 83.3 | - |
| P420L | *fbiC* | 7 | 2.2.1(7) | 1 | 1 | 14.3 | 85.7 | 83.3 | P |
| **W88*** | *ddn* | 6 | 2.2.1(6) | 1 | 1 | 11.1 | 88.9 | 66.7 | - |
| **G71D** | *fgd1* | 6 | 3(6) | 1 | 1 | 66.7 | 0 | 0 | S,P |
| P182L | *fbiB* | 6 | 4.3.4.2.1(3); 6(3) | 2 | 2 | 66.7 | 16.7 | 100 | - |
| M93I | *fgd1* | 6 | 4.9(3); 4.1.2.1(2); 2.2.1(1) | 3 | 3 | 83.3 | 0 | - | - |
| -41G>T | *fbiC* | 6 | 1.2.2(6) | 1 | 1 | 83.3 | 0 | 50 | - |
| I693V | *fbiC* | 6 | 3(6) | 1 | 1 | 83.3 | 16.7 | - | - |
| Q120R | *fbiA* | 6 | 4.8(6) | 1 | 1 | 33.3 | 33.3 | 0 | - |
| L67P | *ddn* | 6 | 4.8(6) | 1 | 1 | 83.3 | 0 | - | S,P |
| Y167H | *fbiC* | 6 | 3(6) | 1 | 1 | 100 | 0 | 0 | P |
| K279E | *fbiC* | 6 | 4.1.2.1(6) | 1 | 1 | 100 | 0 | 0 | - |
| T695K | *fbiC* | 6 | 4.1.2.1(6) | 1 | 1 | 0 | 100 | - | - |
| D224N | *fbiB* | 6 | 2.2.1(6) | 1 | 1 | 33.3 | 16.7 | 100 | - |
| P45L | *ddn* | 5 | 4.4.1.1(3); 3(1); 1.1.1(1) | 3 | 3 | 80 | 0 | 100 | S,P |
| G508S | *fbiC* | 5 | 1.2.2(5) | 1 | 1 | 20 | 80 | 100 | - |
| -23C>T | *ddn* | 5 | 4.8(5) | 1 | 1 | 100 | 0 | 100 | - |
| P607A | *fbiC* | 5 | 4.7(5) | 1 | 1 | 100 | 0 | - | P |
| -6A>C | *ddn* | 5 | 4.8(5) | 1 | 1 | 40 | 60 | 100 | - |
| L326F | *fbiB* | 5 | 4.6.1.1(2); 4.1.2.1(1); 4.1.2(1); 4.8(1) | 4 | 4 | 100 | 0 | - | - |
| A206T | *fbiA* | 5 | 2.2.1(5) | 1 | 1 | 80 | 0 | 66.7 | - |

| | | | | | | | | | |
|---|---|---|---|---|---|---|---|---|---|
| V188I | *fbiA* | 5 | 2.2.1(5) | 1 | 1 | 0 | 80 | 66.7 | - |
| R409C | *fbiB* | 5 | 1.2.1(5) | 1 | 1 | 60 | 0 | 100 | P |
| T455A | *fbiC* | 5 | 3(4); 1.1.1(1) | 2 | 2 | 80 | 0 | 100 | - |
| A132T | *fbiC* | 5 | 2.2.1(5) | 1 | 1 | 40 | 20 | - | - |
| **A835V** | *fbiC* | 5 | 1.1.3(5) | 1 | 1 | 100 | 0 | 0 | - |
| K183T | *fgd1* | 5 | 4.3.3(5) | 1 | 1 | 80 | 0 | 0 | - |
| T302A | *fbiA* | 5 | 2.1(5) | 1 | 1 | 40 | 20 | 80 | - |
| A201P | *fbiA* | 5 | 2.1(5) | 1 | 1 | 80 | 0 | 100 | - |
| 85_87del | *ddn* | 4 | 2.2.1(4) | 1 | 1 | 50 | 0 | 50 | - |
| T302P | *fbiA* | 4 | 2.2.1(4) | 1 | 1 | 0 | 100 | 100 | - |
| 508_509insT | *fgd1* | 4 | 4.1.1.3(4) | 1 | 1 | 100 | 0 | 100 | - |
| A380S | *fbiB* | 4 | 3(4) | 1 | 1 | 75 | 25 | - | S,M |
| Q69R | *fbiB* | 4 | 3(1); 4.2.2(3) | 2 | 2 | 100 | 0 | 0 | - |
| V61I | *fgd1* | 4 | 1.1.3(4) | 1 | 1 | 100 | 0 | 100 | - |
| W139* | *ddn* | 4 | 4.1.2(4) | 1 | 1 | 100 | 0 | - | - |
| P438S | *fbiC* | 4 | 4.3.4.2(4) | 1 | 1 | 100 | 0 | 100 | P |
| D126Y | *fbiC* | 4 | 4.8(4) | 1 | 1 | 75 | 0 | 100 | S,P |
| G168R | *fgd1* | 4 | 4.1.1.1(3); 2.2.2(1) | 2 | 2 | 50 | 50 | 0 | - |
| **D168E** | *fbiC* | 4 | 4.1.2(4) | 1 | 1 | 0 | 25 | 100 | - |
| R72Q | *ddn* | 4 | 4.8(4) | 1 | 1 | 100 | 0 | 100 | - |
| R154H | *fbiC* | 4 | 4.6.1.2(4) | 1 | 1 | 0 | 100 | 100 | S,P |
| I18V | *fbiB* | 4 | 4.6.1.2(4) | 1 | 1 | 25 | 25 | - | - |
| R177H | *fbiA* | 4 | 4.1.2.1(2); 4.5(2) | 2 | 2 | 100 | 0 | 100 | - |
| P438L | *fbiC* | 4 | 4.4.1.1(4) | 1 | 1 | 100 | 0 | 0 | P |
| R45C | *fgd1* | 4 | 4.3.2(4) | 1 | 1 | 0 | 75 | 100 | P |
| V123I | *fbiB* | 4 | 4.6.2.2(4) | 1 | 1 | 0 | 100 | 100 | - |
| 283_303del | *ddn* | 4 | 4.5(4) | 1 | 1 | 100 | 0 | 100 | - |
| 3986845_3987298de l | *ddn* | 4 | 2.2.1(4) | 1 | 1 | 0 | 100 | 100 | - |
| T218A | *fbiC* | 4 | 2.2.1.1(4) | 1 | 1 | 0 | 100 | 100 | - |
| R293W | *fbiB* | 4 | 1.2.1(4) | 1 | 1 | 75 | 0 | 100 | S,P |
| Q170H | *fbiA* | 4 | 1.1.1(3); 1.2.2(1) | 2 | 2 | 100 | 0 | - | P |
| G839D | *fbiC* | 4 | 1.1.1(4) | 1 | 1 | 100 | 0 | 100 | - |
| D263N | *fgd1* | 4 | 1.1.3(4) | 1 | 1 | 50 | 50 | 0 | - |
| V301L | *fbiA* | 4 | 4.4.1.1(4) | 1 | 1 | 100 | 0 | 100 | - |
| D387N | *fbiC* | 4 | 2.2.1(4) | 1 | 1 | 75 | 0 | 100 | - |
| R334Q | *fbiB* | 4 | 1.1.1.1(4) | 1 | 1 | 75 | 0 | 100 | S |
| D78N | *fbiD* | 4 | 4.5(4) | 1 | 1 | 100 | 0 | 100 | - |
| -37T>C | *fbiC* | 3 | 2.2.1.1(3) | 1 | 1 | 100 | 0 | 100 | - |
| G145A | *fgd1* | 3 | 5(3) | 1 | 1 | 33.3 | 33.3 | - | - |
| -38G>A | *fgd1* | 3 | 3(3) | 1 | 1 | 100 | 0 | - | - |
| -3C>T | *fgd1* | 3 | 4.1.2.1(3) | 1 | 1 | 66.7 | 0 | - | - |
| A111T | *ddn* | 3 | 1.2.2(1); 4.5(1); 3(1) | 3 | 3 | 66.7 | 0 | - | S,P |

| | | | | | | | | | |
|---|---|---|---|---|---|---|---|---|---|
| A333V | *fbiC* | 3 | 3.1.2(3) | 1 | 1 | 0 | 66.7 | - | - |
| N556D | *fbiC* | 3 | 1.2.1(2); 3(1) | 2 | 2 | 66.7 | 0 | 100 | S,P |
| G310* | *fbiC* | 3 | 1.2.1(3) | 1 | 1 | 100 | 0 | - | - |
| G70V | *ddn* | 3 | 1.2.2(3) | 1 | 1 | 100 | 0 | - | S,P |
| D542N | *fbiC* | 3 | 4.1.2(3) | 1 | 1 | 100 | 0 | 100 | P |
| V621I | *fbiC* | 3 | 4.8(2); 2.2.1(1) | 2 | 2 | 66.7 | 33.3 | 100 | - |
| V61G | *ddn* | 3 | 4.1.2(3) | 1 | 1 | 66.7 | 0 | - | S |
| -40C>A | *ddn* | 3 | 3(3) | 1 | 1 | 0 | 0 | - | - |
| V241I | *fbiA* | 3 | 5(3) | 1 | 1 | 100 | 0 | 100 | - |
| -33G>A | *fbiC* | 3 | 1.1.1(3) | 1 | 1 | 100 | 0 | - | - |
| V46G | *ddn* | 3 | 5(3) | 1 | 1 | 0 | 100 | - | S,P |
| V740A | *fbiC* | 3 | 4.1.2.1(3) | 1 | 1 | 100 | 0 | 0 | S |
| -31T>C | *fbiC* | 3 | 4.1.2.1(3) | 1 | 1 | 100 | 0 | 0 | - |
| A237V | *fbiB* | 3 | 1.2.2(3) | 1 | 1 | 100 | 0 | 0 | - |
| G26S | *fbiB* | 3 | 4.1.2.1(3) | 1 | 1 | 100 | 0 | 0 | P |
| D406A | *fbiB* | 3 | 4.1.2.1(3) | 1 | 1 | 100 | 0 | 0 | P |
| E474A | *fbiC* | 3 | *Bov*(3) | 1 | 1 | 100 | 0 | 0 | - |
| R137H | *fbiB* | 3 | 4(3) | 1 | 1 | 100 | 0 | 0 | - |
| V389L | *fbiC* | 3 | 4.8(3) | 1 | 1 | 100 | 0 | 0 | - |
| -10G>C | *fbiC* | 3 | 4.3.2(3) | 1 | 1 | 100 | 0 | 0 | - |
| K236N | *fbiC* | 3 | 4.8(3) | 1 | 1 | 100 | 0 | 0 | S,P |
| A77T | *ddn* | 3 | 4.8(3) | 1 | 1 | 100 | 0 | 0 | S,P |
| R330P | *fbiC* | 3 | 1.2.1(3) | 1 | 1 | 100 | 0 | 0 | P |
| -10G>A | *fbiC* | 3 | 4.3.4.2(3) | 1 | 1 | 0 | 100 | - | - |
| A10V | *fgd1* | 3 | 1.1.2(3) | 1 | 1 | 100 | 0 | - | - |
| A206S | *fbiA* | 3 | 2.2.2(3) | 1 | 1 | 100 | 0 | 66.7 | - |
| L723F | *fbiC* | 3 | 2.2.1(1); 4.3.4.2.1(2) | 2 | 1 | 66.7 | 33.3 | 66.7 | - |
| S42G | *fbiA* | 3 | 2.2.2(3) | 1 | 1 | 100 | 0 | - | - |
| A404V | *fbiC* | 3 | 2.2.1(3) | 1 | 1 | 66.7 | 0 | - | - |
| A620T | *fbiC* | 3 | 2.2.1(3) | 1 | 1 | 66.7 | 0 | 100 | - |
| P15S | *fbiC* | 3 | 4.5(3) | 1 | 1 | 0 | 0 | 100 | - |
| 527_534del | *fgd1* | 3 | 2.2.1(2); 4.3.2.1(1) | 2 | 3 | 33.3 | 33.3 | 100 | - |
| G74C | *fbiC* | 3 | 1.2.2(3) | 1 | 1 | 100 | 0 | 100 | P |
| **S78Y** | *ddn* | 3 | 2.2.1(3) | 1 | 1 | 100 | 0 | - | S,P |
| V37G | *fgd1* | 3 | *Bov*(1); 4.1.2(2) | 2 | 2 | 0 | 0 | 100 | S,P |
| A29T | *fgd1* | 3 | 1.1.1(3) | 1 | 1 | 0 | 0 | 100 | - |
| -48C>T | *fbiC* | 3 | 3.1.2(3) | 1 | 1 | 33.3 | 0 | 50 | - |
| -40C>T | *ddn* | 3 | 3.1.2(3) | 1 | 1 | 100 | 0 | - | - |
| **L228F** | *fbiC* | 3 | 3(3) | 1 | 1 | 100 | 0 | 0 | - |
| **K2E** | *fbiA* | 3 | 3(2); 1.1.2(1) | 2 | 2 | 66.7 | 0 | - | - |
| **W88R** | *ddn* | 3 | 3(1); 4.1.1.3(2) | 2 | 2 | 33.3 | 66.7 | 100 | S,P, M |
| V625A | *fbiC* | 3 | 1.2.2(3) | 1 | 1 | 0 | 66.7 | - | - |

| | | | | | | | | | |
|---|---|---|---|---|---|---|---|---|---|
| V155M | *fbiA* | 3 | 3(1); 1.1.1.1(2) | 2 | 2 | 100 | 0 | 100 | - |
| Q27P | *fbiA* | 3 | 4.1.2.1(3) | 1 | 1 | 100 | 0 | - | - |
| P206L | *fbiC* | 3 | 4.8(1); 2.2.1(2) | 2 | 2 | 66.7 | 0 | 100 | P |
| E312K | *fbiC* | 3 | 1.1.3(3) | 1 | 1 | 0 | 100 | - | - |
| <u>A43T</u> | *fbiA* | 3 | 4.4.1.1(2); 1.1.2(1) | 2 | 2 | 66.7 | 0 | 100 | - |
| <u>M319I</u> | *fbiA* | 3 | 2.2.1(3) | 1 | 1 | 0 | 100 | 100 | - |
| P63S | *ddn* | 3 | 1.1.2(3) | 1 | 1 | 100 | 0 | 100 | S,P |
| I246T | *fbiA* | 3 | 4.2.2(3) | 1 | 1 | 100 | 0 | 100 | P |
| I193V | *fgd1* | 3 | 1.1.2(3) | 1 | 1 | 66.7 | 0 | 100 | - |
| I208M | *fbiA* | 3 | 1.1.2(3) | 1 | 1 | 33.3 | 33.3 | 100 | - |
| **Y65S** | *ddn* | 3 | 4.5(3) | 1 | 1 | 0 | 0 | 100 | S |
| P111L | *fbiC* | 3 | 2.2.1(3) | 1 | 1 | 66.7 | 0 | 100 | P |
| E65G | *fbiB* | 3 | 4.8(3) | 1 | 1 | 100 | 0 | 100 | - |
| G8D | *fbiD* | 3 | 2.2.1(3) | 1 | 1 | 33.3 | 66.7 | 50 | - |
| I10V | *fbiD* | 3 | 4.2.2(3) | 1 | 1 | 0 | 0 | 100 | - |
| A20V | *fbiD* | 3 | 2.2.1(3) | 1 | 1 | 100 | 0 | 100 | - |
| T34S | *fbiD* | 3 | 3.1.1(3) | 1 | 1 | 100 | 0 | 0 | - |
| G76S | *fbiD* | 3 | 4.2(3) | 1 | 1 | 100 | 0 | 100 | P |
| E127Q | *fbiD* | 3 | 4.3.4.1(3) | 1 | 1 | 66.7 | 33.3 | - | - |
| G155S | *fbiD* | 3 | 1.2.2(3) | 1 | 1 | 66.7 | 0 | 0 | - |
| V211G | *fbiD* | 3 | 2.2.1(3) | 1 | 1 | 100 | 0 | 100 | - |
| -45G>C | *fbiD* | 3 | 4.5(3) | 1 | 1 | 0 | 100 | - | - |
| -34G>C | *fbiD* | 3 | 3.1.2(3) | 1 | 1 | 100 | 0 | 0 | - |
| T302I | *fbiB* | 2 | 1.2.1(1); 2.2.1(1) | 2 | 2 | 0 | 50 | 100 | - |
| **V154I** | *fbiA* | 2 | 4.2.1(2) | 1 | 1 | 100 | 0 | 100 | - |
| E282D | *fbiB* | 2 | 2.2.1(2) | 1 | 1 | 100 | 0 | - | - |
| -17T>TC | *ddn* | 2 | 4.1.1.3(2) | 1 | 1 | 100 | 0 | 100 | - |
| 381_464del | *fbiA* | 2 | 4.1.2.1(1); 1.2.2(1) | 2 | 2 | 100 | 0 | - | - |
| K282N | *fbiC* | 2 | 1.1.2(2) | 1 | 1 | 100 | 0 | - | - |
| V16F | *fbiC* | 2 | 1.2.2(2) | 1 | 1 | 0 | 0 | 100 | - |
| H364Y | *fbiC* | 2 | 3(1); 4.1.2.1(1) | 2 | 2 | 50 | 0 | - | P |
| G755S | *fbiC* | 2 | 3(1); 4.1.1.3(1) | 2 | 2 | 50 | 0 | - | P |
| I262V | *fgd1* | 2 | 4.1.1.3(2) | 1 | 1 | 100 | 0 | - | M |
| E608K | *fbiC* | 2 | 3(2) | 1 | 1 | 0 | 0 | - | - |
| R780C | *fbiC* | 2 | 4.8(2) | 1 | 1 | 100 | 0 | - | P |
| V25A | *fgd1* | 2 | 4.9(2) | 1 | 1 | 0 | 0 | - | M |
| G277S | *fbiA* | 2 | 3(2) | 1 | 1 | 100 | 0 | - | P |
| <u>V41M</u> | *fbiC* | 2 | 1.1.2(2) | 1 | 1 | 100 | 0 | - | - |
| G293A | *fbiA* | 2 | 4.1.2.1(2) | 1 | 1 | 100 | 0 | - | - |
| E332K | *fbiB* | 2 | 4.4.1.2(2) | 1 | 1 | 50 | 0 | - | - |
| A10T | *fgd1* | 2 | 3.1.2(2) | 1 | 1 | 100 | 0 | - | M |
| 273_273del | *ddn* | 2 | 3(2) | 1 | 1 | 100 | 0 | - | - |
| P78S | *fbiC* | 2 | 4.6.2(1); 2.1(1) | 2 | 2 | 50 | 50 | 100 | - |

| | | | | | | | | | |
|---|---|---|---|---|---|---|---|---|---|
| R99W | *fbiC* | 2 | 4.1.2.1(1); 4.3.4.1(1) | 2 | 2 | 100 | 0 | - | S,P |
| D308G | *fbiC* | 2 | 3(2) | 1 | 1 | 100 | 0 | - | P |
| R458H | *fbiC* | 2 | 1.1.3(1); 6(1) | 2 | 2 | 100 | 0 | - | P |
| P370R | *fbiC* | 2 | 3(1); 4.4(1) | 2 | 2 | 100 | 0 | - | P |
| R365G | *fbiB* | 2 | 3(2) | 1 | 1 | 100 | 0 | 100 | P,M |
| I247N | *fbiA* | 2 | 4.7(2) | 1 | 1 | 100 | 0 | - | P |
| G94R | *fgd1* | 2 | 4.1.2.1(2) | 1 | 1 | 100 | 0 | - | P |
| E127D | *fbiC* | 2 | 4.1.2.1(2) | 1 | 1 | 0 | 0 | - | - |
| M268V | *fgd1* | 2 | 4.7(2) | 1 | 1 | 100 | 0 | - | - |
| <u>S184T</u> | *fbiA* | 2 | 3(2) | 1 | 1 | 50 | 50 | - | - |
| **P361A** | *fbiB* | 2 | 4(2) | 1 | 1 | 100 | 0 | - | P |
| G541S | *fbiC* | 2 | 4.1.2.1(1); 1.2.2(1) | 2 | 2 | 50 | 50 | - | P |
| L93F | *fbiB* | 2 | 4.3.2.1(1); 4.8(1) | 2 | 2 | 50 | 50 | - | - |
| A404P | *fbiC* | 2 | 4.3.3(2) | 1 | 1 | 100 | 0 | - | - |
| G78S | *fbiA* | 2 | 4.3.4.2(1); 4.3.4.2.1(1) | 2 | 2 | 50 | 0 | 100 | S,P |
| <u>D66E</u> | *fbiB* | 2 | 4.3.4.2.1(2) | 1 | 1 | 100 | 0 | 100 | - |
| <u>D465A</u> | *fbiC* | 2 | 4.6(2) | 1 | 1 | 100 | 0 | - | P |
| G159V | *fgd1* | 2 | 4.1.2.1(2) | 1 | 1 | 100 | 0 | 100 | - |
| H295R | *fbiA* | 2 | 4.1.2.1(2) | 1 | 1 | 0 | 50 | 100 | - |
| P193S | *fbiC* | 2 | 4.1.2.1(2) | 1 | 1 | 100 | 0 | - | P |
| R845C | *fbiC* | 2 | 6(1); 1.1.1(1) | 2 | 2 | 100 | 0 | 100 | P |
| T185A | *fbiC* | 2 | 6(2) | 1 | 1 | 50 | 0 | 100 | P |
| I816V | *fbiC* | 2 | 5(2) | 1 | 1 | 100 | 0 | - | - |
| G445D | *fbiC* | 2 | 3(2) | 1 | 1 | 50 | 50 | 100 | P |
| T292A | *fbiB* | 2 | 4.3.3(2) | 1 | 1 | 0 | 100 | 100 | P |
| <u>P16R</u> | *fbiB* | 2 | 2.2.1(2) | 1 | 1 | 100 | 0 | 100 | S,P |
| A136S | *fbiC* | 2 | 4.8(1); 4.1.2(1) | 2 | 2 | 50 | 50 | 100 | - |
| <u>D69N</u> | *ddn* | 2 | 4.3.2.1(1); 4.3.4.2(1) | 2 | 2 | 0 | 100 | 100 | - |
| G264E | *fbiA* | 2 | 1.2.1(1); 4.4.2(1) | 2 | 2 | 50 | 0 | 50 | S,P |
| <u>F220L</u> | *fbiB* | 2 | 4.1.2.1(1); 4.8(1) | 2 | 2 | 100 | 0 | 0 | - |
| T268I | *fbiB* | 2 | 2.2.1(1); 3(1) | 2 | 2 | 50 | 50 | 100 | - |
| R321S | *fbiA* | 2 | 3(2) | 1 | 1 | 0 | 100 | 100 | - |
| G512C | *fbiC* | 2 | 4.3.3(2) | 1 | 1 | 100 | 0 | 100 | - |
| D147N | *fbiA* | 2 | 4.6.1.1(2) | 1 | 1 | 0 | 0 | - | P |
| N66Y | *fgd1* | 2 | 2.2.1(2) | 1 | 1 | 50 | 50 | 100 | - |
| R486H | *fbiC* | 2 | 4.5(1); 4(1) | 2 | 2 | 50 | 50 | - | - |
| R68H | *ddn* | 2 | 2.2.1(2) | 1 | 1 | 100 | 0 | 0 | - |
| R550C | *fbiC* | 2 | 2.2.1(2) | 1 | 1 | 100 | 0 | 100 | S,P |
| -46GGTGGGGC>G | *fbiC* | 2 | 2.2.2(2) | 1 | 1 | 100 | 0 | - | - |
| -9T>C | *fbiC* | 2 | 1.1.1(2) | 1 | 1 | 100 | 0 | - | - |
| V390G | *fbiB* | 2 | 2.2.2(1); 3(1) | 2 | 2 | 50 | 0 | - | - |

| | | | | | | | | | |
|---|---|---|---|---|---|---|---|---|---|
| -18T>C | *fgd1* | 2 | 4.8(2) | 1 | 1 | 100 | 0 | - | - |
| N32T | *ddn* | 2 | 4.2.1(2) | 1 | 2 | 0 | 100 | 100 | - |
| *152G | *ddn* | 2 | 4.5(2) | 1 | 1 | 100 | 0 | 100 | - |
| -22C>T | *fbiA* | 2 | 4.5(2) | 1 | 1 | 100 | 0 | 100 | - |
| F306V | *fbiC* | 2 | 4.4.2(2) | 1 | 1 | 100 | 0 | 100 | - |
| V33I | *fbiB* | 2 | 2.2.1(2) | 1 | 1 | 100 | 0 | 100 | - |
| L374S | *fbiC* | 2 | 2.2.1(2) | 1 | 1 | 0 | 100 | 100 | S,P |
| -17A>C | *fbiC* | 2 | 4.4.2(2) | 1 | 1 | 0 | 100 | 100 | - |
| P362S | *fbiC* | 2 | 2.2.1(2) | 1 | 1 | 0 | 100 | 100 | P |
| E13G | *fbiB* | 2 | 2.2.1.1(2) | 1 | 1 | 0 | 100 | 100 | - |
| I167V | *fbiA* | 2 | 2.2.1(2) | 1 | 1 | 0 | 100 | 100 | - |
| H183N | *fbiA* | 2 | 2.2.1(2) | 1 | 1 | 0 | 100 | 100 | P |
| T796A | *fbiC* | 2 | 2.2.2(2) | 1 | 1 | 0 | 100 | 100 | - |
| R212Q | *fgd1* | 2 | 1.1.1.1(1); 7(1) | 2 | 2 | 100 | 0 | 100 | - |
| D74E | *fbiA* | 2 | 1.2.2(2) | 1 | 1 | 100 | 0 | - | - |
| G189D | *fbiA* | 2 | 2.2.1(2) | 1 | 2 | 100 | 0 | - | P |
| M313L | *fbiB* | 2 | 2.2.1(2) | 1 | 1 | 100 | 0 | 0 | - |
| P6L | *ddn* | 2 | 2.2.1(2) | 1 | 1 | 0 | 50 | - | P |
| L204F | *fbiC* | 2 | 2.2.1(2) | 1 | 1 | 0 | 50 | - | - |
| D203N | *fbiB* | 2 | 1.1.1(2) | 1 | 1 | 100 | 0 | - | P |
| D148N | *fbiA* | 2 | 1.1.1(2) | 1 | 1 | 50 | 0 | - | P |
| A178G | *fbiA* | 2 | 2.2.1(2) | 1 | 1 | 100 | 0 | - | - |
| P60S | *fbiA* | 2 | 3(2) | 1 | 1 | 0 | 100 | 0 | P |
| E205K | *fgd1* | 2 | 1.2.2(2) | 1 | 1 | 0 | 0 | - | - |
| A63T | *fbiC* | 2 | 1.1.2(1); 2.2.1(1) | 2 | 1 | 50 | 50 | 100 | - |
| S132C | *ddn* | 2 | 1.2.1(2) | 1 | 1 | 50 | 0 | - | - |
| V147M | *ddn* | 2 | 3(2) | 1 | 1 | 100 | 0 | - | - |
| A856T | *fbiC* | 2 | 4.8(2) | 1 | 1 | 100 | 0 | - | - |
| V581I | *fbiC* | 2 | 4.1.2.1(1); 2.2.1(1) | 2 | 2 | 50 | 50 | - | - |
| H46D | *fgd1* | 2 | 3(1); 4.8(1) | 2 | 2 | 50 | 0 | - | P |
| E83A | *ddn* | 2 | 4.2.1(2) | 1 | 1 | 0 | 0 | 0 | - |
| -29C>G | *fgd1* | 2 | 1.1.2(1); 3(1) | 2 | 2 | 50 | 0 | - | - |
| A659V | *fbiC* | 2 | 3(2) | 1 | 2 | 50 | 50 | - | - |
| T50I | *ddn* | 2 | 4.8(2) | 1 | 2 | 100 | 0 | - | S,P |
| I638L | *fbiC* | 2 | *Bov*(2) | 1 | 1 | 100 | 0 | - | - |
| G839S | *fbiC* | 2 | 3(1); *Bov*(1) | 2 | 2 | 50 | 0 | - | - |
| A328V | *fbiB* | 2 | *Bov*(2) | 1 | 1 | 0 | 0 | - | - |
| T36P | *fgd1* | 2 | 2.2.1(2) | 1 | 1 | 100 | 0 | 100 | - |
| M709I | *fbiC* | 2 | 2.2.1(2) | 1 | 1 | 0 | 100 | - | S |
| 851_939del | *fgd1* | 2 | 4.7(1); 2.2.1(1) | 2 | 2 | 0 | 50 | - | - |
| E105Q | *ddn* | 2 | 1.1.2(1); 4.1.2.1(1) | 2 | 2 | 50 | 50 | 50 | - |
| V599A | *fbiC* | 2 | 4.2.2(2) | 1 | 1 | 50 | 0 | 50 | - |
| R134L | *fbiC* | 2 | 3(1); 2.1(1) | 2 | 2 | 100 | 0 | 100 | - |

| | | | | | | | | | | |
|---|---|---|---|---|---|---|---|---|---|---|
| A212P | *fbiA* | 2 | 4.6(1); 4.3.3(1) | 2 | 2 | 0 | 100 | - | P |
| R139Q | *fbiB* | 2 | 4.3.2.1(2) | 1 | 1 | 100 | 0 | 100 | - |
| V495A | *fbiC* | 2 | 3.1.1(2) | 1 | 1 | 100 | 0 | 100 | - |
| E526K | *fbiC* | 2 | 4.3.4.2.1(2) | 1 | 1 | 100 | 0 | 100 | - |
| G114S | *fbiB* | 2 | 4.3.4.2.1(2) | 1 | 1 | 100 | 0 | 100 | - |
| I102V | *ddn* | 2 | 1.2.2(1); 4.1.2.1(1) | 2 | 2 | 50 | 0 | 100 | - |
| A34T | *fbiB* | 2 | 1.1.3(2) | 1 | 1 | 100 | 0 | 100 | - |
| S87L | *fbiC* | 2 | 4.3.3(2) | 1 | 1 | 100 | 0 | - | S,P |
| T185I | *fbiC* | 2 | 4.7(2) | 1 | 1 | 0 | 100 | 100 | S,P |
| E342Q | *fbiC* | 2 | 1.2.2(2) | 1 | 1 | 0 | 50 | 100 | - |
| A518G | *fbiC* | 2 | 1.1.2(2) | 1 | 1 | 100 | 0 | 100 | - |
| P272S | *fbiB* | 2 | 1.1.2(2) | 1 | 1 | 100 | 0 | 100 | - |
| A82T | *fbiB* | 2 | 4.3.2.1(1); 1.1.1(1) | 2 | 2 | 100 | 0 | 100 | - |
| E299V | *fbiC* | 2 | 2.2.1(2) | 1 | 1 | 0 | 0 | 100 | - |
| -6G>T | *fgd1* | 2 | 2.2.1.1(2) | 1 | 1 | 100 | 0 | 100 | - |
| T374K | *fbiB* | 2 | 2.2.1(2) | 1 | 1 | 0 | 50 | 100 | S,P |
| T371A | *fbiB* | 2 | 1.1.1.1(2) | 1 | 1 | 100 | 0 | 100 | - |
| V136M | *fgd1* | 2 | 2.2.1(2) | 1 | 1 | 0 | 100 | 100 | - |
| 490706_490745del | *fgd1* | 2 | 4.7(2) | 1 | 1 | 0 | 100 | 100 | - |
| P270L | *fbiB* | 2 | 4.1.2.1(2) | 1 | 1 | 100 | 0 | - | P |
| I10T | *fbiD* | 2 | *Bov*(2) | 1 | 1 | 0 | 0 | 0 | M |
| V16I | *fbiD* | 2 | 1.2.1(2) | 1 | 1 | 50 | 0 | 100 | - |
| A21T | *fbiD* | 2 | 4.1.1.3(2) | 1 | 1 | 50 | 0 | 100 | - |
| A22T | *fbiD* | 2 | 4.1.1.3(2) | 1 | 1 | 100 | 0 | - | S |
| T48I | *fbiD* | 2 | 4.8(2) | 1 | 1 | 100 | 0 | - | - |
| G106E | *fbiD* | 2 | *Bov*(2) | 1 | 1 | 0 | 0 | 0 | - |
| V111I | *fbiD* | 2 | 1.1.3(2) | 1 | 1 | 50 | 50 | 100 | - |
| T122P | *fbiD* | 2 | 2.2.2(2) | 1 | 1 | 0 | 100 | 100 | - |
| I129M | *fbiD* | 2 | 2.2.1(2) | 1 | 1 | 0 | 100 | 0 | - |
| C187F | *fbiD* | 2 | 4.3.3(2) | 1 | 1 | 0 | 100 | 100 | - |
| -40C>A | *fbiD* | 2 | 3(2) | 1 | 1 | 100 | 0 | 0 | - |
| -39G>T | *fbiD* | 2 | 3(2) | 1 | 1 | 100 | 0 | - | - |

Bedaquiline (BDQ), delamanid (DLM); pretomanid (PTM); Sub-lineages: $^{+}$ = more than 1 sub-lineage; # = number; Drug resistance (%): Susc. = Susceptible; * % of number of samples pre-2014/total number of samples with available collection date; ** Functional support: S = snap2 score >= 50; P = Provean Score =< -4; M = mCSM predicted stability change (ΔΔG) below -2; mutations associated with increased minimum inhibitory concentration for DLM or PTM in previous studies in bold; mutations associated with susceptibility to MIC underlined (see **S3 Table**).

**S7 Table.** Mutations observed in single isolates in the 33k dataset.

| Drug* | Gene | Mutation |
|---|---|---|
| **BDQ** | *atpE* | A6V, G13S, I16V, *M17I* (B), A18S, *I26V* (B), V30I, *E44A* (B), *F50L* (B), *P52L* (B), ***I66V*** (B), -40G>GT, -39C>T, -8A>AT, -9GAT>G, -28TACCAGAGCC>T, -32C>T, -33A>G, -39C>G, 225_226insTTCGCTACACCCGTCAAGTAA |
| **BDQ** | *mmpR5* | **S2I**, V7F, D8G, E13A, E13*, D15A, D15G, E18D, *G24S* (S), ***G25D*** (M), G25S, Y26C, E28A, S29F, S29C, **W42***, E49K, *Q51P* (S), *Q51H* (S), A61T, S64R, *G65W* (S,P), G66V, *S68I* (S,P), **R72Q**, M73I, ***L74P*** (S,P,M), Q76E, *G78V* (S,P), V85F, A86V, 87GDRRTYFRLRPN>87GGSAHLFPVAAH, D88G, *R89W* (S,P), *R89L* (P), R89Q, *L95S* (M), ***R96W*** (S,P), **R96Q**, P97T, N98K, **A99V**, A101S, A102T, G103S, E104G, R107G, R109Q, A110V, ***M111K*** (S), A112T, Q115P, R134G, ***L136P*** (S,P), R137Q, *V149G* (M), A153V, ***L154P*** (S), R160Q, 17_18insGGT, **30_30del**, 70_71insGC, 107_108insG, 113_131del, **138_139insGA**, 139_140insATC, **212_212del**, 216_309del, 234_235insT, 285_285del, **289_289del**, 429_429del, 430_431insCA, 431_432insT 462_462del, 479_480insA, 778997_779279del, -3C>A, -10A>C, -22A>C, -30C>G, -31A>G, -33G>T, -46G>A |
| **BDQ** | *pepQ* | Q13E, S25R, I28V, *Y32H* (S,P), *S39F* (P), *N40S* (P), *G41R* (S), V45L, F46L, A47G, S66P, L71V, E72D, V73M, A78V, V79A, G80R, A84V, G88S, G91D, G93R, *F97V* (P), H100R, *T103M* (P), V104M, V104L, G106S, A109V, K117R, N118D, E120D, L121V, T127S, E148G, A152V, V158M, R160C, R160P, R170W, V172M, R174S, A178D, M180V, D182E, *E191V* (S,P), *E191G* (S,P), A196T, A201G, *R206W* (P), R206L, *T208I* (P), A227D, *M233T* (P), V238M, D244N, Y250H, *R261G* (P), A263V, R271Q, A284G, *F290V* (S,P), *F290L* (S,P), Q301R, G309E, T315A, S320F, S320C, R333H, A345V, K350Q, E368K, A370T, A370V, L372V, 138_139insTC, 947_948insG, 2859300_2860417del |
| **DLM/ PTM** | *ddn* | **M1T**, L13R, S14N, K19R, R23Q, T26I, *W27G* (S), W27C, **W27***, R31S, R31C, G34E, G36V, *G38R* (S,P), K43E, *T51A* (P), T52N, **G53D**, *G53S* (S,P), *R54G* (S,P), *R54C* (S,P), Q58K, Q58P, **Q58***, *P59Q* (P), *N62K* (S), G71R, *V75A* (S,M), *K79E* (S), M87I, N91T, *N95K* (P), K97N, V98F (S,P), V100I, *Q101P* (S,P), K104R, E105K, E117K, P124S, L126F, M129T, M129I, **Y133C**, Y133*, *Y133H* (S,P), Q137R, T140I, -1C>T, -3G>A, -4C>T, -5G>A, -11G>A,-26G>A, -32T>G, -32T>C, -34C>T, -39G>A, -39GC>G, -44C>T, 6_7insAAATC, 24_29del, 36_36del, 59_101del, 90_90del,92_92del, 164_165del, 211_211del, 255_260del, 267_267del, 270_281del, 285_285del, 309_310insT, 312_312del, 322_323insA, 323_330del, 367_369del, 451_455del, 3986810_3986932del, 3986856_3987298del, 3986857_3987298del, |
| **DLM/ PTM** | *fgd1* | *L4R* (P), *S11P* (P), Q14R, A16T, E19D, V21I, A26T, M32V, V37F, Q47L, G62A, N66S, *T76I* (P), T78I, *F79S* (S,P,M), V85I, T92S, C95Y, T107A, T107I, A115S, *Y118S* (M), E119G, F129V, A130T, A130G, R131Q, G137R, Q141H, *D146G* (P), D146N, D153A, D153E, S161L, *I162T* (M), V165L, D167E, V172I, *A182V*, Y184C (P), A188G, E201G, E201K, L202P (P), E205D, K206T, *P209A* (P), A210G, E213K, A218T, D219N, R220Q, K227R, **E230K**, S234A, P237T, P239S, N244E, N245S, N245D, *P251L* (P), T255I, A256P, Q258K, K259E (S), S261N, E267K, A272T, L275P (P), V286M, *P290L* (P), A293V, T302R, *F320L* (P), Q325E, *P330L* (P), R331S, -4A>C, -22G>A, -33G>A, -40G>C, -42G>T, -45CG>C, 502_504del, 643_648del, 986_986del, 490706_490720del |
| **DLM/ PTM** | *fbiA* | T4N, A7G, *G12S* (P), *R14H* (P), L22V, L25M, A30T, S32P, S35P, S35A, A37V, S42C, A43G, I53V, I53L, I53T, V58I, *G71S* (P), *R77H* (P), *R77L* (P), Q81R, D83N, *W101R* (S,P), A121V, *Y123S* (P), *Y123C* (P), *P124R* (P), L125V, ***S126P*** (S,P), T129S, A131T, D134N, *P138L* (P), *G139D* (P), D158N, K165T, A166V, A178S, Q179K, *P181S* (P), *G189S* (P), S194N, A196V, **I209V**, V218I, *A232E* (P), A238T, *P245S* (S,P), **K250***, |

| | | |
|---|---|---|
| | | *M255T* (P), D266N, A269S, A271T, *G277D* (P), A278G, C280R, C287Y, V290M, G293S, A296V, D299A, *M310T* (S,P), V303M, A315V, A322V, A331G, -8C>A, -42C>T, 196_283del, 866_866del |
| **DLM/ PTM** | *fbiB* | S8F, E13K, *G19W* (S,P), *E22G* (P), *R24L* (P), *G26R* (P), *P38Q* (P), *P38A* (P), *K51N* (S,P), *E57A* (P), *R59W* (S,P), L60M, *P64A* (P), D66N, Q69P, E79R, A104T, *A105T* (S), G114D, A119T, A127T, T131I, L132V, G135E, G141S (P), V142I, A145T, *Q160H* (P), V165I, A171S, R180C, E186A, E186D, V188M, V192I, G221S, V222A, D225N, N238D, L243F, A246S, E247D, R253H, *R260L* (S,P), V263I, *R265W* (S), *P270Q* (P), V280I, *H290Y* (S,P), R293Q, V298M, S323N, D324E, P327L, A328T, A328S, *R334G* (S,P), *R334W* (S,P), *G338S* (P), D343E, A344V, E346Q, I349V, I349M, *A357E* (P), A364T, T366N, T366I, A368T, E369Q, *G394S* (S,P), S395N, *I402T* (P), *R409H* (P), D410G, D410H, *P415L* (P), L435S, *P438S* (P), V439A, P440S, A441T, K448E, 1175_1266del |
| **DLM/ PTM** | *fbiC* | V1L, G6S, V16I, V17L, P18A, P19R, A21P, A25T, *R27W* (S,P), R31Q, A33T, A33V, V37A, V37G, A45T, A47T, T49A, C59Y, *R75W* (S,P), *F91C* (S,P), *P93L* (S,P), *P93S* (P), *R96L* (P), *R96G* (S,P), ***C105R*** (S,P), *L114R* (P), T121A, D126E, D130N, D130E, R134Q, A136V, E137Q, *F145L* (P), *T146S* (S), *R150S* (S,P), E152D, A153V, *R159G* (S,P), E160D, *E160G* (P), D168A, *S169F* (S,P), S172C, S172F, A208V, *M211I* (S), *R221Q* (S), *D237G* (P), *P238L* (P), A239E, *R243S* (P), *T257A* (S,P), *E269G* (S,P), *D272G* (S,P), L274F, H275Y, *R278L* (S,P), H281Q, K282R, *R296H* (P), A302V, A305V, F306L, *P307S* (P), I311V, *D313G* (S,P), Y314F, A321T, *P327L* (P), R330S, *P334A* (P), *P334S* (P), G340R, D341E, C343W, *R344W* (S), D375N, *D375G* (P), ***L377P*** (P), M388V, M388L, Q395H, Q400R, A401G, V410M, *R411W* (S), R411S, A418E, *P420S* (P), G423S, D427H, *W435G* (P), P438R, V441A, A442V, S443C, *R446W* (P), Q456E, *R458P* (P), V462L, *R463C* (P), *D472A* (P), V495L, V495M, L496R, A497T, A497P, D511E, A516T, T519N, T519I, *G522R* (P), *G522S* (P), *G522A* (P), A527S, V540A, *F554L* (S,P), T560A, K571E, *R587W* (S,P), A588V, M601I, *M601T* (S,P), I605V, D606E, *P610S* (P), T612A, A620V, N640T, N640S, *G646E* (P), S648T, *W652R* (S,P), I654V, E658D, T663N, *D674H* (P), *P686T* (P), L689W, T695M, *G711A* (S,P), A721V, N724K, I729V, R732H, *R732L* (S,P), *G734S* (P), *H746Y* (P), Q747H, *P750R* (P), *L753R* (P), A756V, *R758C* (S,P), *P759R* (P), *P759T* (P), *H770Y* (P), *G792A* (S,P), E801N, *G808D* (S,P), M812V, *E813A* (P), E813D, T815A, E823G, E823Q, H824Y, A827S, *G841R* (P), *P843Q* (P), *P843A* (P), *R845H* (P), L853P, A855L, **A856P**, *857W, 24_25insTCCACCGCTCTGCCGAGTCCC,342_347del, 785_786insCAT, 897_898del, 1132_1133insCTT, 1133_1134insTTT, 1205_1206del, 1223_1252del, 1651_1731del, 1871_1871del, 2162_2162del, 2331_2335del, 2545_2546insTCACATACGCCCTGCTTGC, 2551_2552insACGCC, 2562_2623del, 1305491_1305500del, -3A>G, -13A>AC, -14G>A, -17A>G, -18C>A, -23G>A, -27A>G, -29A>C, -29A>G, -30G>C, -32A>C, -33G>C, -41G>A, -46G>C, -46GGT>G, -49C>G |
| **DLM /PTM** | *fbiD* | P5L, I13V, *P28L* (P), F30L, *S31W* (P), V38L, V39A, V44I, A50T, A51S, G52S, V53G, I62V, E66Q, A70T, A81P, P85A, A98T, A99T, R101C, A104V, E105A, G106V, L144R, T146S, V150I, H159N, *R186C* (S,P), V189I, A206T, A210S, -21C>T, -24G>A, -31C>T, -38A>G, -45G>A |

* Bedaquiline (BDQ), Delamanid (DLM); Pretomanid (PTM) has similar resistance mechanisms to DLM. In bold, known resistant mutations (see **S3 Table**); underlined: known susceptible mutations (see **S3 Table**); italic: with one parameter predicting to have a functional effect: B = SUSPECT-BDQ predicted as resistant (only for *atpE*); S = snap2 score >= 50; P = Provean score =< -4; M = mCSM predicted stability change (ΔΔG) below -1. There is no prediction for indels or variants in the promoter region.

**S8 Table.** Loss of function mutations in the *ndh* gene.

| Mutation | Frequency | Lineage | Resistance profile * |
|---|---|---|---|
| Q4* | 1 | 1 (1.1.2) | S |
| Y56* | 1 | 3 | XDR |
| Q57* | 2 | 1 (1.1.1) | S |
| Y112* | 1 | 1 (1.1.2) | S |
| C273* | 1 | 3 | XDR |
| 970_971insGG | 8 | 2(n=5;2.2.1); 4(4.1.2.1(n=2),4.3.4.2(n=1) | MDR(4), XDR(4) |
| 304_304del | 82 | 2(2.2.1.1) | MDR(76), XDR(5), DR(1) |
| 149_158del | 1 | 1(1.2.1) | MDR |
| 972_973inC | 1 | 2(2.2.1) | XDR |
| 970_971insG | 2 | 2(2.2.1),4(4.3.3) | XDR,DR |
| 1120_1120del | 1 | 1(1.1.1) | DR |
| 965_965del | 2 | 2(2.2.1) | MDR |
| 15_15del | 2 | 4(4.2.2) | MDR |
| 838_838del | 1 | 1(1.2.1) | MDR |
| 902_903insG | 2 | 2(2.2.1) | MDR,XDR |
| 1007_1008insGC | 1 | 4(4.4.2) | MDR |
| 941_942insGGGTA | 1 | 2(2.2.1) | XDR |
| 1347_1348insA | 1 | 4(4.1.2.1) | S |
| 293_294insG | 6 | 4(4.1.2.1) | XDR |
| 330_337del | 1 | 4(4.3.1) | XDR |
| 900_901insC | 1 | 2(2.2.1) | MDR |
| 633_634insTG | 1 | 1(1.2.1) | DR |
| 199_200insG | 1 | 4(4.1.2.1) | XDR |
| 760_761insC | 1 | 2(2.2.1) | MDR |
| 1206_1207insCG | 1 | 4(4.9) | MDR |
| 2098715_2102885del | 1 | 2(2.2.1) | XDR |
| 2102284_2103965del | 3 | 2(2.2.1) | XDR |
| 2098094_2101927del | 1 | 4(4.3.3) | DR |
| 2096545_2103436del | 1 | 1(1.2.1) | S |
| 2098085_2104480del | 1 | 2(2.2.1) | XDR |
| 2097074_2107484del | 1 | 2(2.2.1) | MDR |
| 2102559_2103251del | 1 | 1(1.1.2) | S |

* Resistance profile: DR = Drug-resistant, S = Susceptible

**S9 Table. Phenotypic data from the Portuguese *M. tuberculosis* isolates**

| Isolate | *mmpR5*/Rv0678 Mutation[a] | MIC (mg/L) | | Resistance Type | Phenotypic Drug Resistance[b] | Genotype[c] |
|---|---|---|---|---|---|---|
| | | BDQ | CFZ | | | |
| **MTB1** | Ile67fs | 0.25 | 1 | XDR | $INH^R$ $RIF^R$ $STR^R$ $EMB^R$ $PZA^R$ $AMK^S$ $CAP^R$ $KAN^R$ $CIP^R$ | L4.3.4.2/SIT20/LAM1/Lisboa3 |
| **MTB2** | WT | ≤0.015 | 0.125 | XDR | $INH^R$ $RIF^R$ $STR^R$ $EMB^R$ $PZA^R$ $AMK^S$ $CAP^S$ $KAN^R$ $CIP^R$ | L4.3.4.2/SIT20/LAM1/Lisboa3 |
| **MTB3** | WT | ≤0.015 | 0.25 | Susceptible | Pan susceptible | L4.3.4.1/SIT17/LAM2/NC |
| **MTB4** | WT | ≤0.015 | 0.25 | MDR | $INH^R$ $RIF^R$ $STR^R$ $EMB^R$ $PZA^R$ $AMK^R$ $CAP^R$ $KAN^R$ $CIP^S$ | L4.3.4.2/SIT1106/LAM4/Q1 |
| **MTB5** | WT | 0.03 | 0.25 | MDR | $INH^R$ $RIF^R$ $STR^S$ $EMB^S$ $PZA^S$ $AMK^S$ $CAP^S$ $KAN^S$ $CIP^S$ | L4.1.2.1/SIT53/T1/NC |
| **H37Rv (ATCC 27294)** | WT | 0.03 | 0.25 | Susceptible (Reference Strain) | Pan susceptible | - |

[a] WT – wildtype allele for *mmpR5*/Rv0678;

[b] R and S in superscript denotes phenotypic resistance or susceptibility to given drug, respectively. INH, isoniazid; RIF, Rifampicin; STR, streptomycin; EMB, ethambutol; PZA, pyrazinamide; AMK, amikacin; CAP, capreomycin; KAN, kanamycin; CIP, ciprofloxacin;

[c] NC – non-clustered isolate.

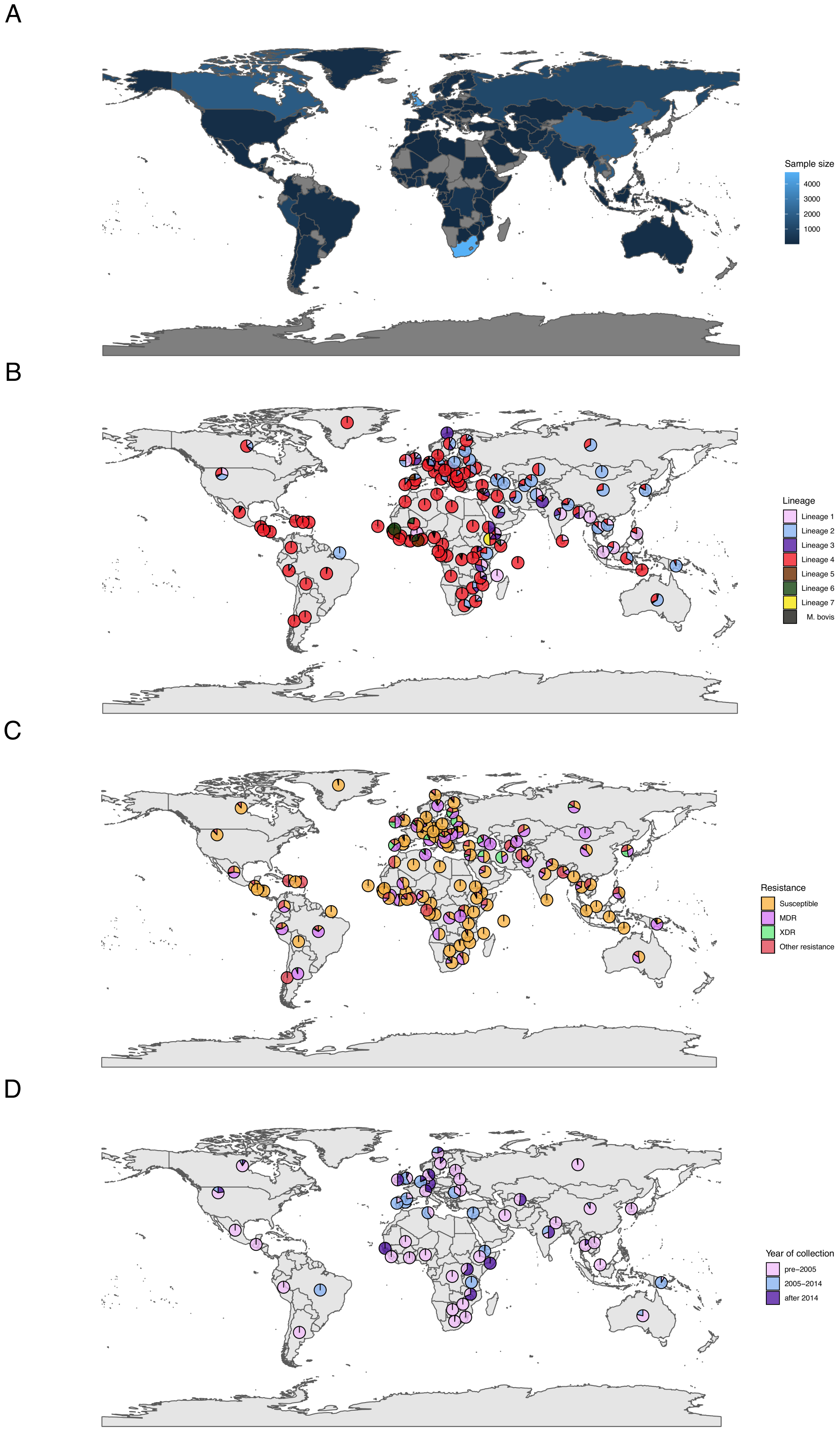


**S1 Figure.** The isolates analysed by country: **(A)** Sample size; **(B)** Lineage; **(C)** Resistance; **(D)** Year of collection. The R (v3.4.3) statistical package was used to generate the maps (https://www.r-project.org).

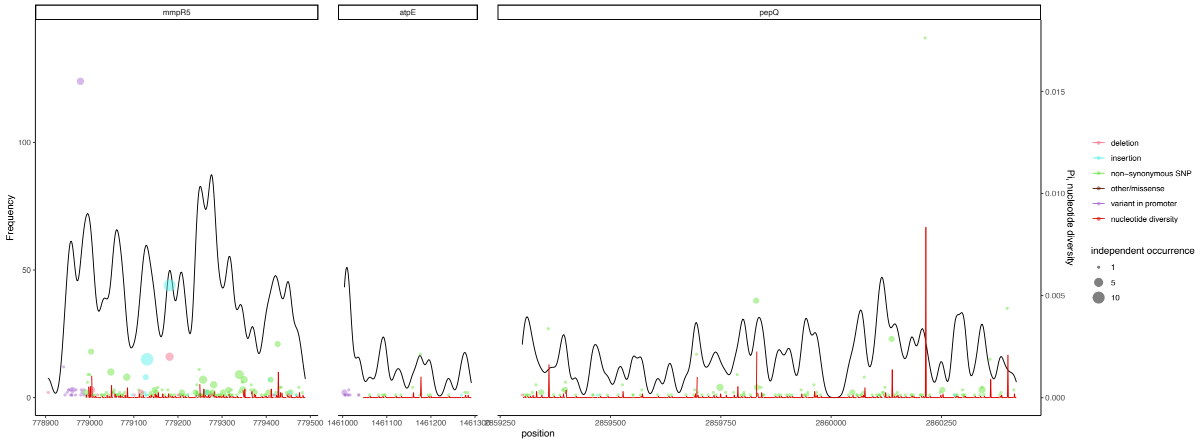


**S2 Figure.** Density of mutations and nucleotide diversity (Nei's Pi) along BDQ resistance genes. Density line is represented in black. Nucleotide diversity (only non-synonymous SNPs) by position (Nei's Pi) is represented in red. Left vertical axis is frequency of each mutation represented by a point (type of mutation differ in colour), and size represents the independent occurrence of each mutation in the phylogenetic tree.

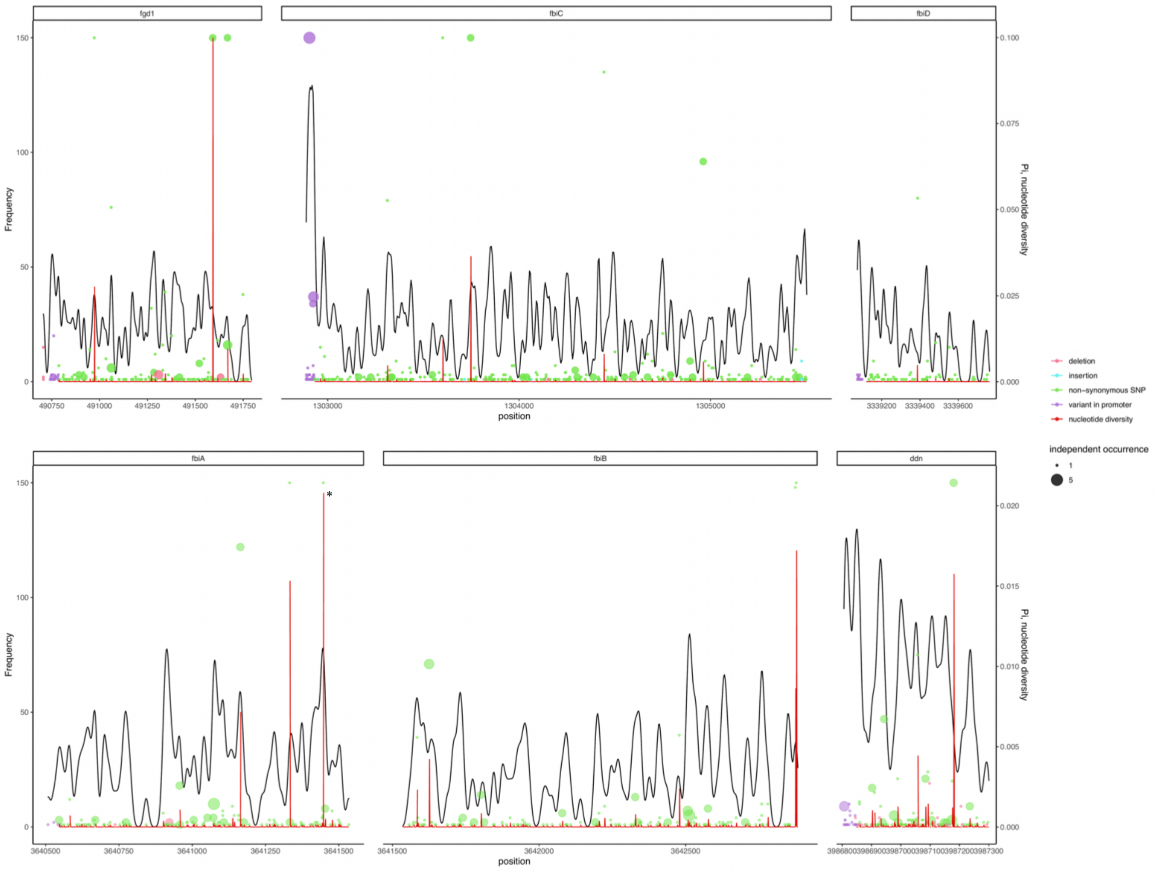


**S3 Figure.** Density of mutations and nucleotide diversity (Nei's Pi) along Delamanid (DLM) and Pretomanid (PTM) resistance genes. Density line is represented in black. Nucleotide diversity (only non-synonymous SNPs) by position (Nei's Pi) is represented in red. Left vertical axis is frequency of each mutation represented by a point (type of mutation differ in colour), and size represents the independent occurrence of each mutation in the phylogenetic tree; * Nucleotide diversity at position 491592 in *fgd1* is 0.168; ** Mutations with frequency >150 have been represented at 150.

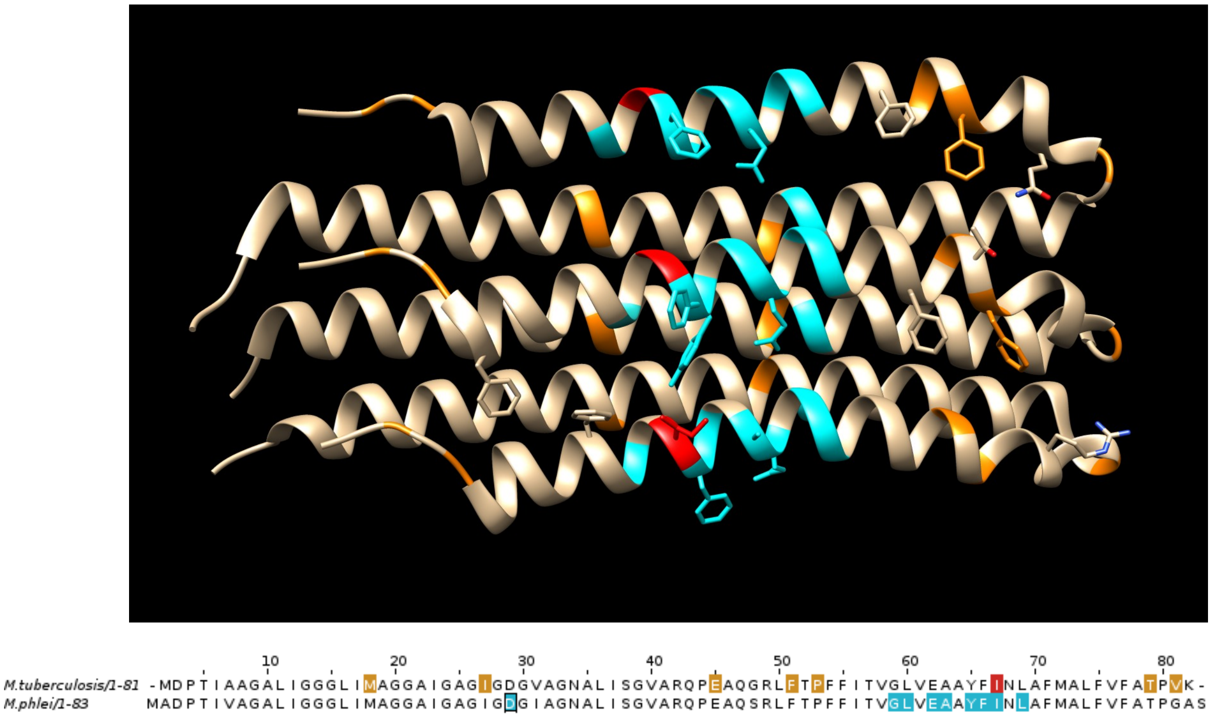


**S4 Figure.** Protein structure of *atpE* C9 ring and sequence. The c9 ring is composed by 3 subunits. Highlighted in blue are the residues known to interact with Bedaquiline (BDQ), in orange the residues predicted to give resistance, and in red the known and previously reported mutation associated with BDQ drug resistance.

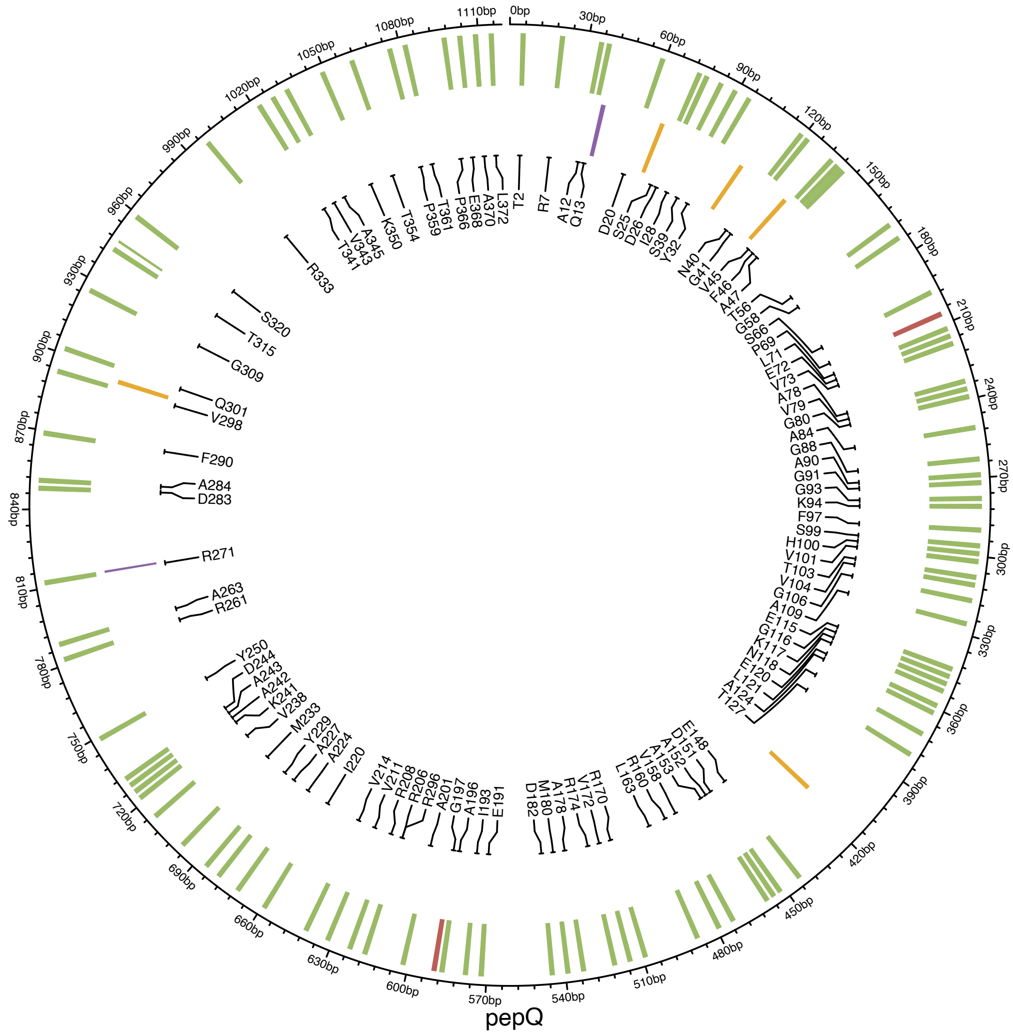


**S5 Figure.** Non-synonymous SNPs and indels in the *pepQ* gene, a candidate for bedaquiline (BDQ) resistance. From outside to inside, first track represents indels (in red) and SNPs (in green) identified in the ~33k isolates. SNPs leading to premature stop codons in blue. The second track represents known resistant SNPs (yellow) and indels (purple). Labels show the residues where SNPs are identified in the ~33k isolates: in black residues with not known association to susceptibility/resistance; in green residues with known association to susceptibility; in red residues with known association to increased minimum inhibitory concentration (MIC) values.

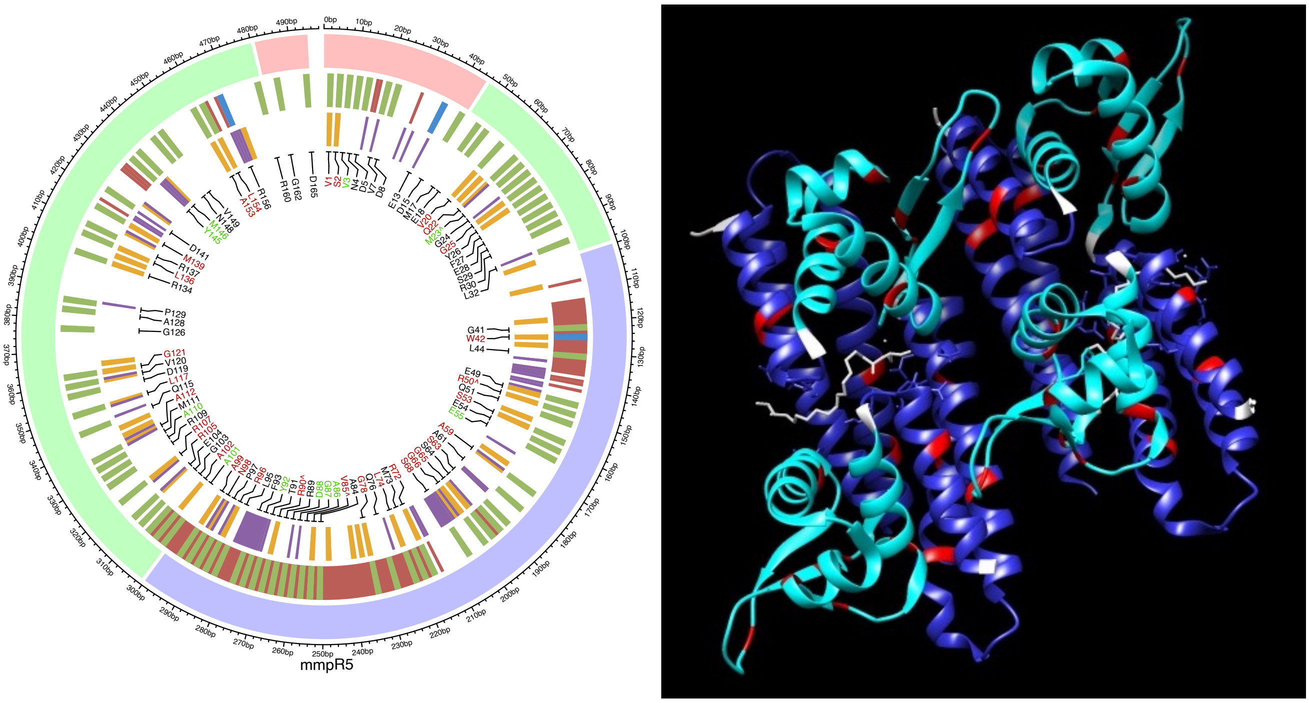


**S6 Figure.** *MmpR5* variants position and protein structure. **(left)** Non-synonymous SNPs and indels along *ddn* gene. From outside to inside, first track represents the different domains of the protein: in red non-characterised; in green dimerization domain; in blue binding domain. The second track show indels (in red) and SNPs (in green) identified in the ~33k isolates. SNPs leading to premature stop codons in blue. The third track represents known resistant SNPs (yellow) and indels (purple). Labels show the residues where SNPs are identified in the ~33k isolates: in black residues with not known association to susceptibility/resistance; in green residues with known association to susceptibility; in red residues with known association to increased minimum inhibitory concentration; ^ = residues with association to resistance and susceptibility depending on alternate allele. Non-synonymous SNPs and indels position along the *mmpR5* gene. SNPs are coloured in green, indels in red. **(right)** Protein structure of *mmpR5* showing in red SNPs that have already seen reported as associated with bedaquiline resistance. Dark blue corresponds to the binding domain.

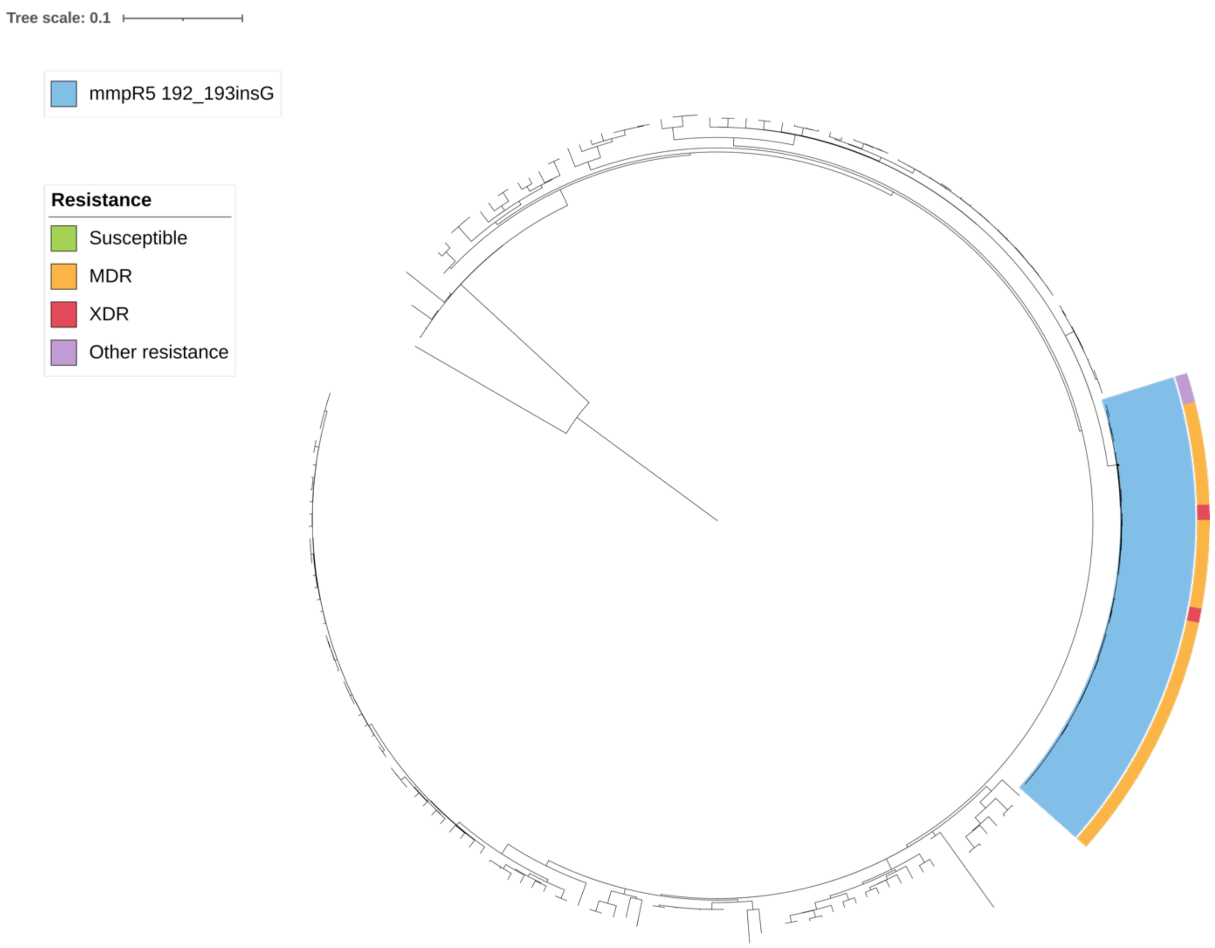


**S7 Figure.** Phylogenetic tree of lineage 4 strains. Coloured in blue are the samples that present the frameshift (192_193insG; I67F) in *mmpR5* for bedaquiline resistance. The outer track shows the resistance profile of the samples harbouring the frameshift.

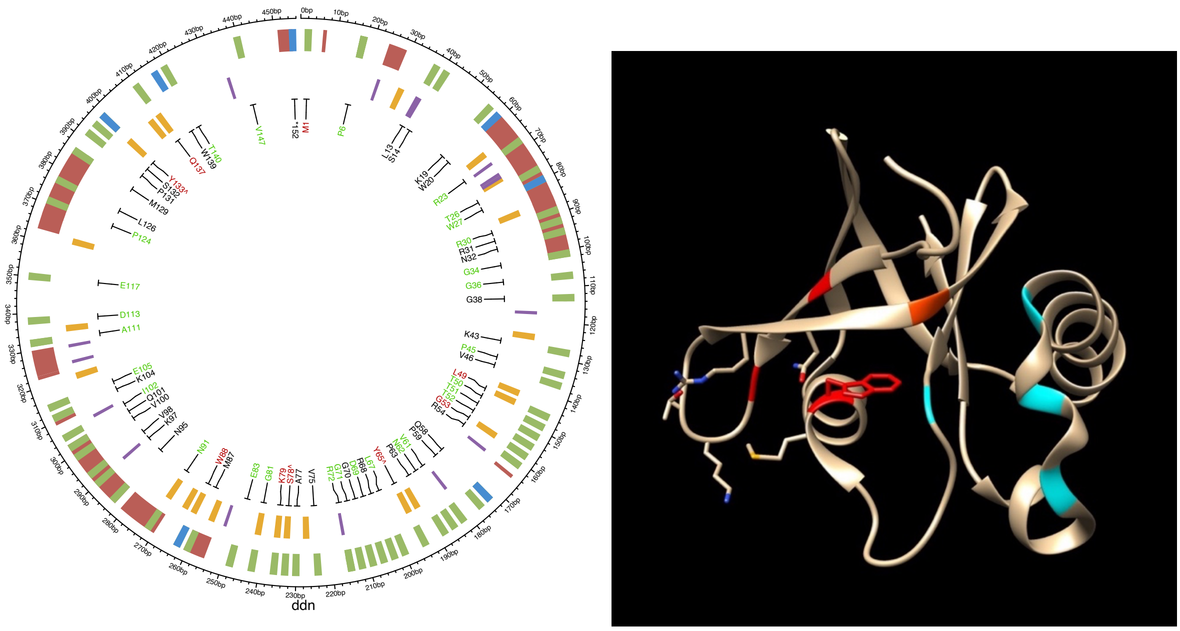


**S8 Figure. A)** Non-synonymous SNPs and indels along *ddn* gene. From outside to inside, first track represents indels (in red) and SNPs (in green) identified in the ~33k isolates. SNPs leading to premature stop codons in blue. The second track represents known resistant SNPs (yellow) and indels (purple). Labels show the residues where SNPs are identified in the ~33k isolates: in black residues with not known association to susceptibility/resistance; in green residues with known association to susceptibility; in red residues with known association to increased MIC; ^ = residues with association to resistance and susceptibility depending on alternate allele or drug (delamanid (DLM)/pretomanid (PTM)). **B)** Protein structure of *ddn* gene showing in red SNPs that have already seen reported as associated with DLM/PTM resistance, in blue residues known to be involved in PTM interaction, and in orange residues involved in PTM interaction that also confer resistance to DLM.

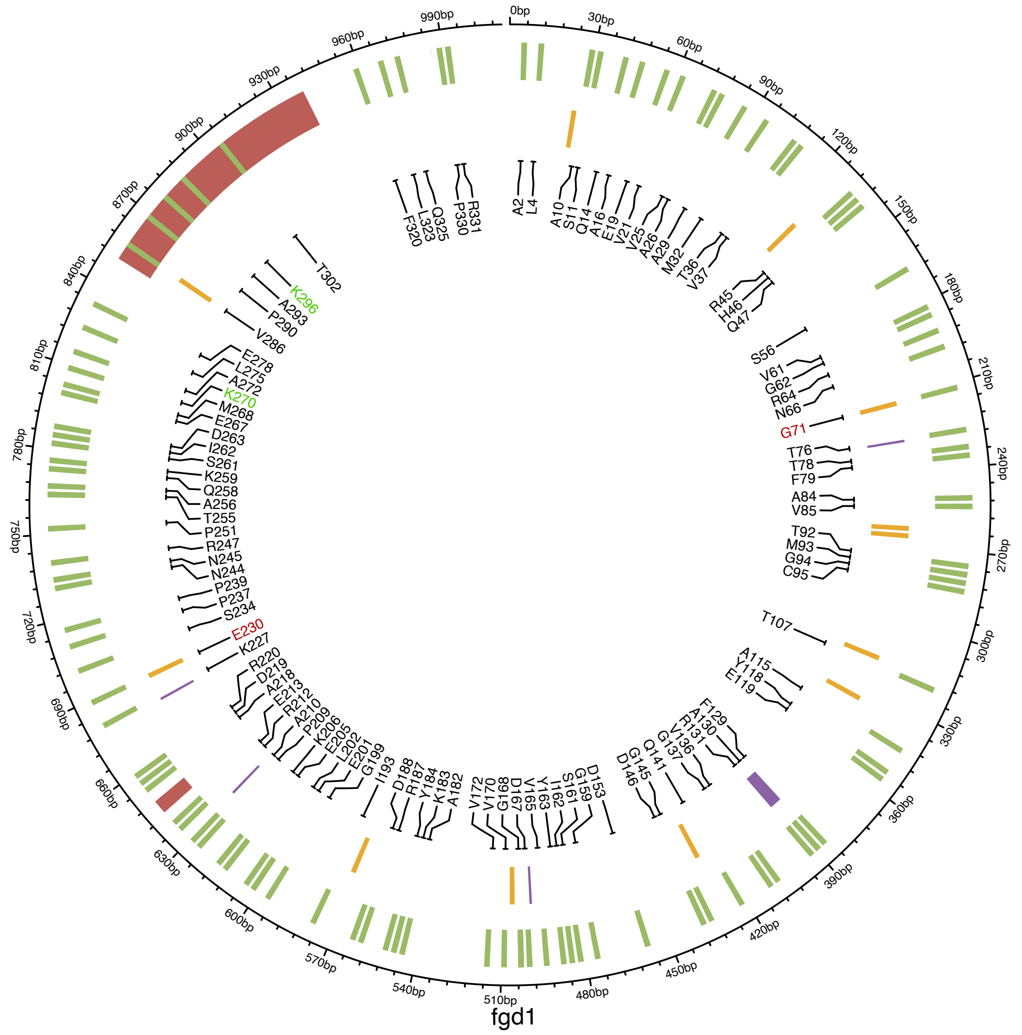


**S9 Figure.** Non-synonymous SNPs and indels in the *fgd1* gene, a candidate for delamanid/pretomanid resistance. From outside to inside, first track represents indels (in red) and SNPs (in green) identified in the ~33k isolates. SNPs leading to premature stop codons in blue. The second track represents known resistant SNPs (yellow) and indels (purple). Labels show the residues where SNPs are identified in the ~33k isolates: in black residues with not known association to susceptibility/resistance; in green residues with known association to susceptibility; in red residues with known association to increased MIC values.

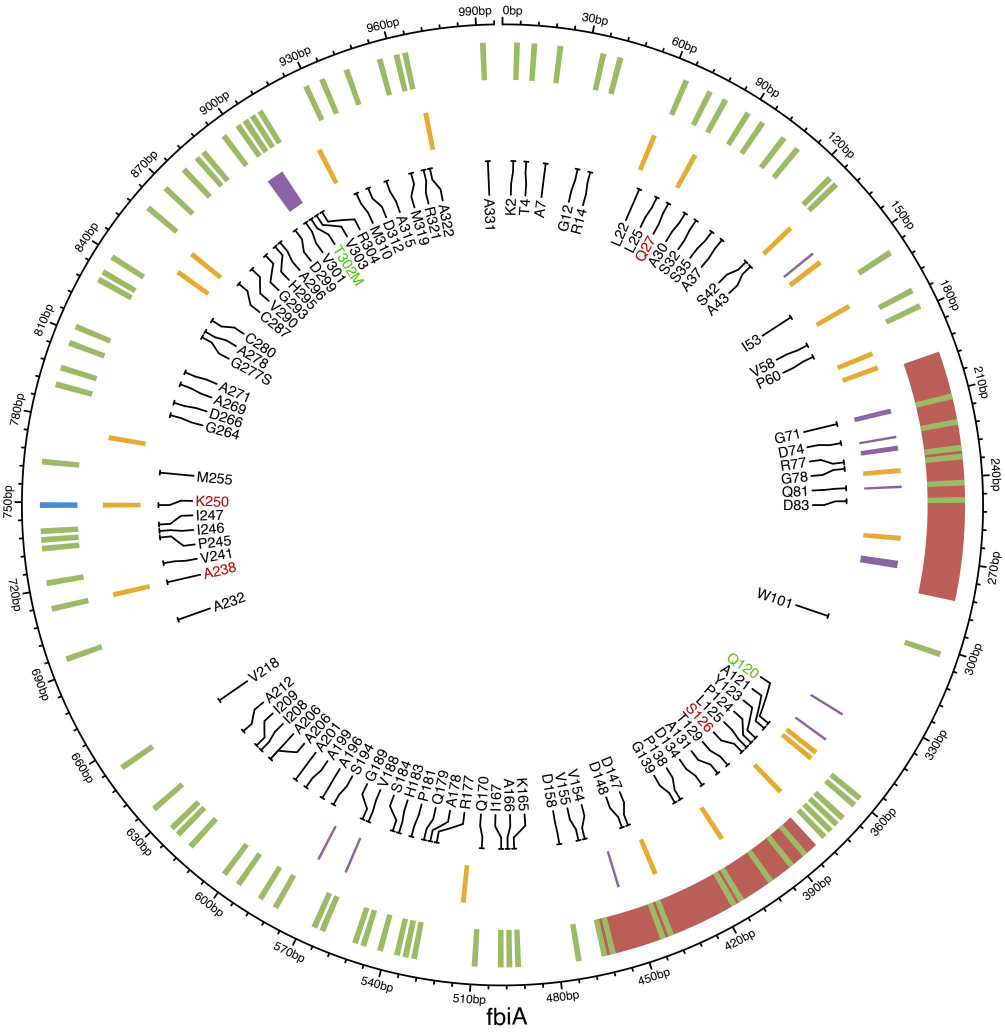


**S10 Figure.** Non-synonymous SNPs and indels in the *fbiA* gene, a candidate for delamanid/pretomanid resistance. From outside to inside, first track represents indels (in red) and SNPs (in green) identified in the ~33k isolates. SNPs leading to premature stop codons in blue. The second track represents known resistant SNPs (yellow) and indels (purple). Labels show the residues where SNPs are identified in the ~33k isolates: in black residues with not known association to susceptibility/resistance; in green residues with known association to susceptibility; in red residues with known association to increased MIC.

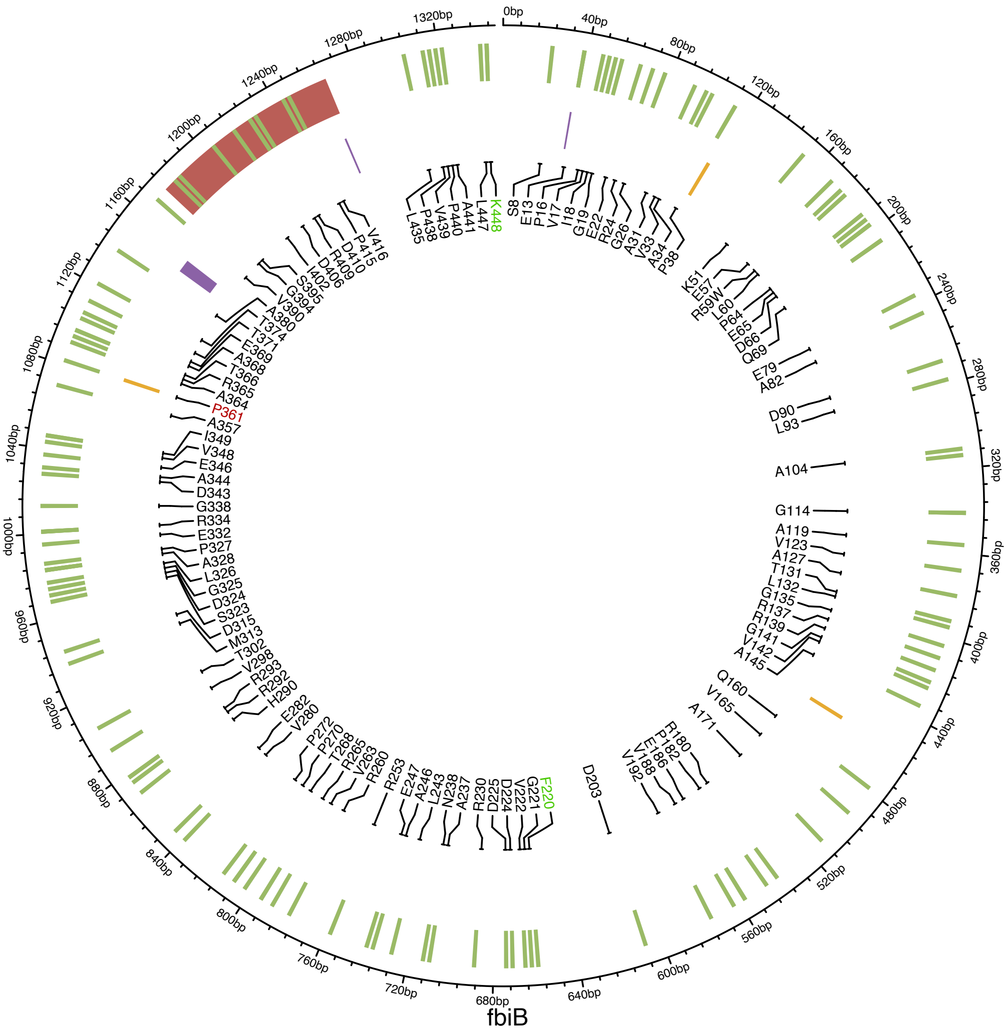


**S11 Figure.** Non-synonymous SNPs and indels in the *fbiB* gene, a candidate for delamanid/pretomanid resistance. From outside to inside, first track represents indels (in red) and SNPs (in green) identified in the ~33k isolates. SNPs leading to premature stop codons in blue. The second track represents known resistant SNPs (yellow) and indels (purple). Labels show the residues where SNPs are identified in the ~33k isolates: in black residues with not known association to susceptibility/resistance; in green residues with known association to susceptibility; in red residues with known association to increased MIC.

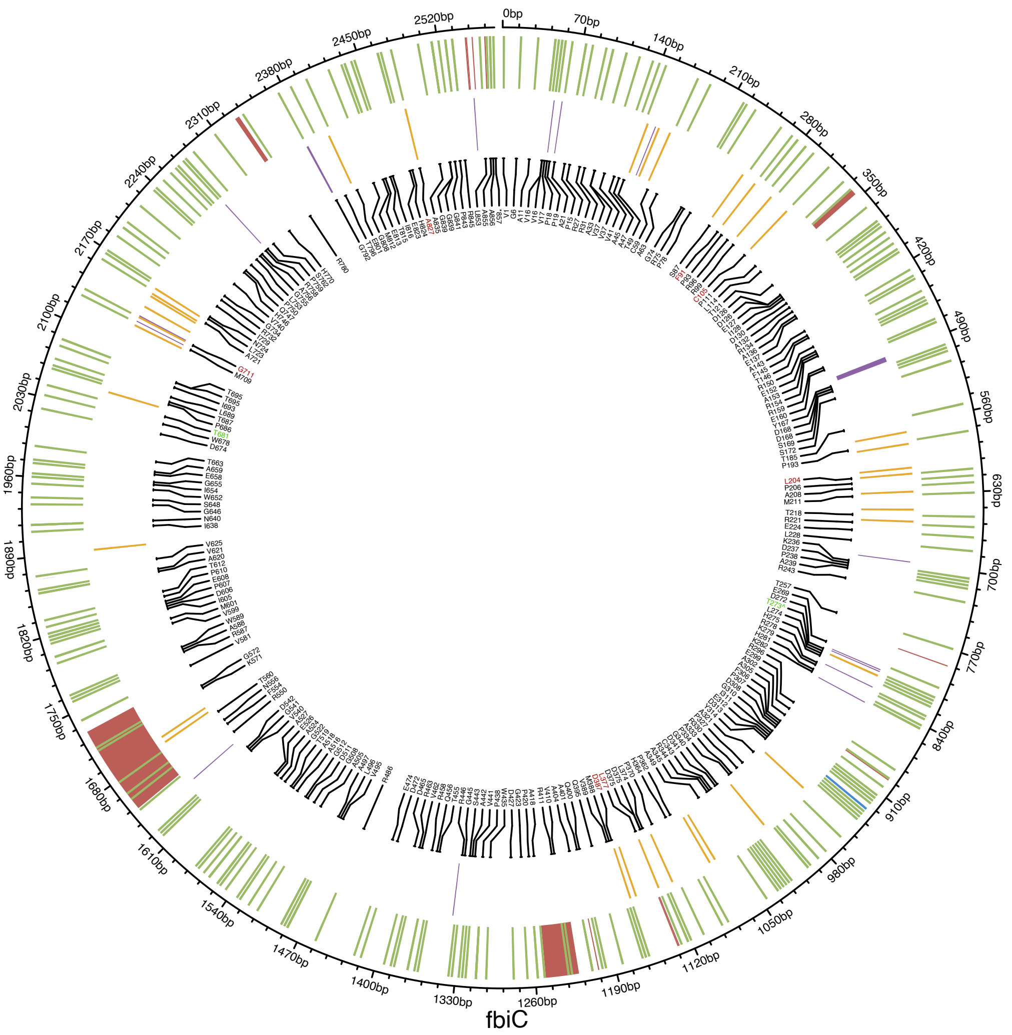


**S12 Figure.** Non-synonymous SNPs and indels in the *fbiC* gene, a candidate for delamanid (DLM)/pretomanid (PTM) resistance. From outside to inside, first track represents indels (in red) and SNPs (in green) identified in the ~33k isolates. SNPs leading to premature stop codons in blue. The second track represents known resistant SNPs (yellow) and indels (purple). Labels show the residues where SNPs are identified in the ~33k isolates: in black residues with no known association to susceptibility/resistance; in green residues with known association to susceptibility; in red residues with known association to increased MIC; ^ = residues with association to resistance and susceptibility depending on alternate allele or drug (DLM/PTM).
